# Magnetic nanoparticle-catalysed synthesis of quinoline derivatives: A green and sustainable method

**DOI:** 10.1016/j.heliyon.2024.e40451

**Published:** 2024-11-20

**Authors:** Vaishali, Shubham Sharma, Pooja Sharma, D.K. Das, Vinod K Vashistha, Jitender Dhiman, Rachna Sharma, Rajesh Kumar, Man vir Singh, Yogendra Kumar

**Affiliations:** a Department of Chemistry, Birla Institute of Higher Education, Pilani, Rajasthan, 333031, India; b Department of Chemistry, GLA University, Mathura, UP, 281406, India; c Department of Chemistry, Lovely Professional University, Jalandhar, Phagwara, Punjab, 144001, India; d Department of Chemistry, University of Lucknow, UP, India; e Central Instrumentation laboratory, Central Pulp and Paper Research Institute, Saharanpur, Uttar Pradesh, India; f Department of Applied Science, TULA’S Institute Dehradun, Uttarakhand, 248197, India; g Department of Chemistry, S.S.J. University Campus Almora, Uttarakhand, 263601, India; h Department of Chemistry, Dev Bhoomi Uttarakhand University, Dehradun, Uttarakhand, 248007, India; i Department of Chemistry, University of Zululand, Corner Guldengracht &, 2 Cent Cir, Road, Richards Bay, 3900, South Africa

**Keywords:** Green catalyst, Magnetic nanoparticles, Inorganic-dopped, Organic-dopped, Quinoline

## Abstract

Greener and sustainable synthetic strategies have been evolving as the demanding domain of organic synthesis during the last decade. Green synthesis involves the development of method that decrease or eliminate the use of hazardous chemicals, and make use of renewable or recyclable resources. By incorporating the fundamentals and methodologies of green synthesis, organic chemists have the ability to develop valuable organic molecular frameworks which also demonstrate a strong commitment to environmental sustainability. In this context, the nanoparticle has garnered significant interest due to its various features, adhering to the principles of green synthesis. Specifically, magnetic nanoparticles have been trending extensive uses in green synthesis throughout the past decade. The role of magnetic nanoparticle has an irreplaceable place in the synthesis of biologically valuable frameworks named as quinoline. Quinoline are considered a privileged structure among organic compounds and offer a promising avenue for identifying lead structures in the search of new synthetic molecules (Saquinavir, Imiquimod and Reabamipide) having potential medicinal values and other important prospects. So, it’s always indeed to the organic and medicinal chemist to develop biologically active frameworks by the green synthesis. The current manuscript consolidates the existing research on properties of environment-friendly magnetic nanoparticles for generating an extended range of valuable quinoline derivatives.

## Graphical abstract

**Figure:**
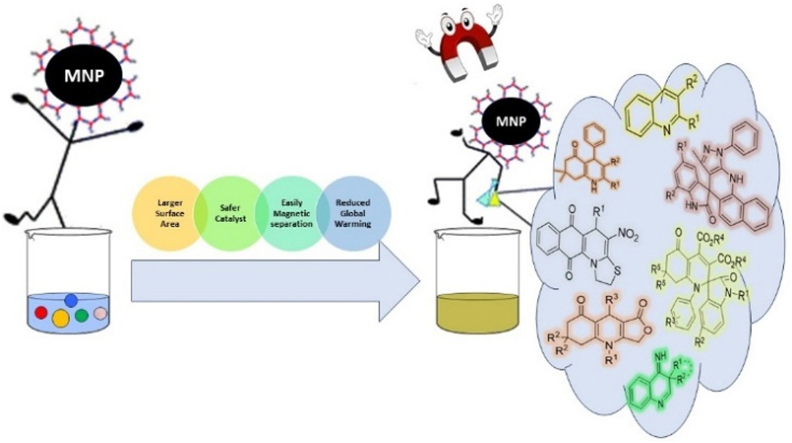

## 1 Introduction

Scientists are well engaged to offer substantial efforts to develop eco-friendly sustainable methods for synthesizing the series of different valuable motifs, following the principles of green chemistry and synthesis [1,2]. To be a successful catalyst according to green chemistry, it needs to maintain low cost, significant stability and selectivity along with reusability demonstrating reasonably high reactivity [3]. Therefore, the development of a flexible catalyst, appropriate for the diverse reactions is definitely a distinct and intricate endeavour. In these perspectives, nanoparticles have been proved its potential as a catalyst to accomplish the different type of organic transformation or reactions [4].

**Fig. 1:**
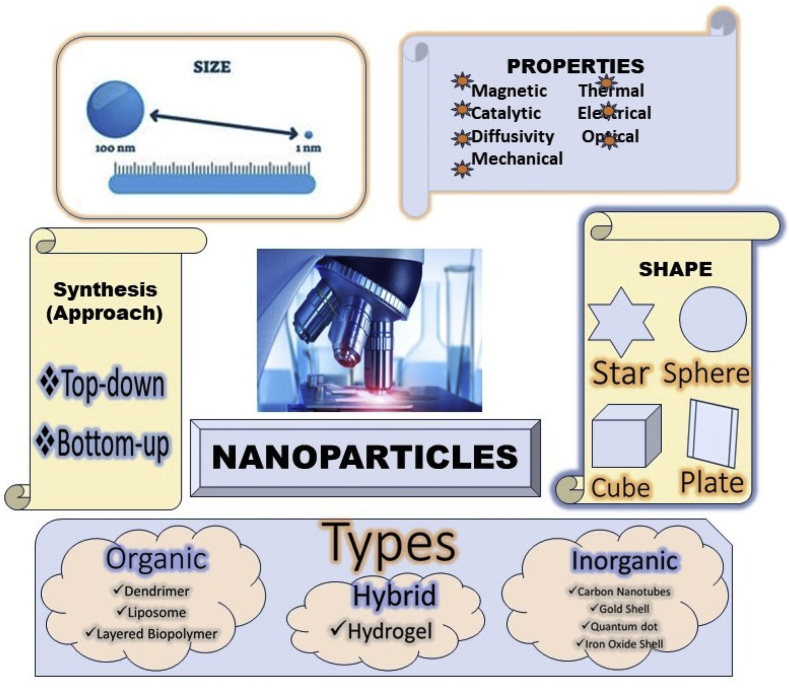
Key points about the nanoparticles in various respect.

Directed towards the field of nano catalyst, homogeneous and heterogenous nanoparticles have several potential characteristics, like enhanced activity, a large surface area, excellent selectivity, stability, and the ability to be recovered and reused [5,6] (Fig. 1). From homogeneous and heterogenous nanoparticles, the extraction of homogeneous nanoparticles from the reaction mixture poses difficulties, thus requiring sophisticated protocol involving filtration or centrifugation and this procedure is frequently challenging, arduous, and time taking as well. In this context, the selection of heterogeneous catalysts is highly sought because of their effortless separation, recoverability, and reusability, which has garnered growing interest [[7], [8], [9], [10], [11], [12], [13], [14], [15], [16], [17]].

Among heterogenous catalyst, utilization of magnetic nanoparticles (Fe_3_O_4_, Fe_2_O_3_, CoFe_2_O_4_, NiFe_2_O_4_, and CuFe_2_O_4_) (MNPs) has been suggested [7]. Particularly, in comparison of non-magnetic heterogenous nanoparticles, the effortless extraction of the heterogenous magnetic nanoparticles from the reaction mixture can be conveniently separated *via* external magnet [8]. Moreover, these magnetic nanoparticles can be utilized repeatedly without any deterioration in their effectiveness [[9], [10], [11], [12], [13], [14], [15], [16], [17], [18], [19], [20]]. On the basis of its profile, magnetic nanoparticle has an irreplaceable place in the field of organic synthesis to act as green, novel and valuable catalyst to develop a important class of organic compounds [21,22].

**Fig. 2:**
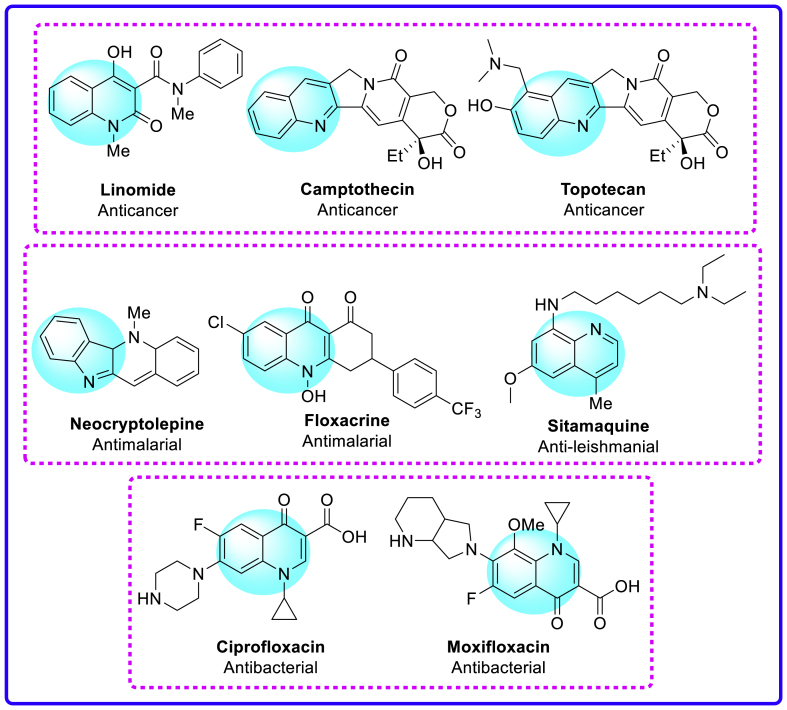
Selected examples of quinoline based bioactive molecular hybrids.

On the other hand, quinoline remains one of the most studied aza-heterocyclic frameworks in the synthetic organic chemistry. This situation owes its origin to the wide-applications of quinoline-derivatives in pharmaceutical, agricultural, and electronic industry [[23], [24], [25], [26], [27]]. In particular, they possess Antimalarial [28,29], Anti-inflammatory [30,31], Antiasthmatic, Antibacterial [32], Antiarrhythmic, Antileishmanial [33], Antitubercular [34,35] and Anticancer [36] activities. Further, quinoline nucleus being present in several alkaloids including Linomide, Camptothecin, and Topotecan *etc.* are known to have remarkable anticancer properties [[37], [38], [39], [40], [41]]. Quinoline based drugs exemplified as Neocryptolepine, Floxacrine, Sitamaquine, and Ciprofloxacin, belong to the members of the class of Antimalarial, Antileishmanial and Antibiotics [42]. Similarly, several other quinoline containing natural products have been a source of inspiration for the scientists while designing new quinoline frameworks for different drug discovery programs [[43], [44], [45]]. Fig. 2 represents the few bioactive quinoline based framework.

Although quinoline derivatives and magnetic nanoparticles have significant importance and distinct reactivity, the synthetic capabilities of quinoline derivatives employing magnetic nanoparticles have not been previously consolidated. Exploring magnetic nanoparticles to their full potential in synthesizing quinoline derivatives has the potential to yield far more promising and commendable outcomes. Thus, the newer and novel synthetic methodologies of quinoline have drawn the attention of synthetic organic or medicinal chemists as evident from the growing numbers of publications in this field. The current review focuses on synthetic methodologies carried out in the last decade with the inherent objective to attain sustainability towards the synthesis of quinoline and their analogues. These findings focus on the practical synthetic methods utilized to create quinoline-based fused and tethered scaffolds, utilizing magnetic nanoparticles. Moreover, this review article highlights the first consolidated findings (from 2008 to present) on the development of quinoline derivatives by the catalysis of magnetic nanoparticles and is likely to be beneficial to the experts working in this field for future prospective.

## 2 Synthesis of quinoline tether/fused derivatives through the magnetic nanoparticles

This section describes the role of magnetic nanoparticles towards the development of biologically active quinoline based scaffolds. For better understanding of the review, we have categorized the whole review in the different section based on the type of nanoparticles. The subsections are mentioned herein.

### 2.1 Role of pure nanoparticles in the synthesis of quinoline derivatives

Particularly, this part illustrates the role of pure nanoparticles for the synthesis of quinoline based tethered/fused molecular architectures. The details study by the various research groups across the globe have presented in easy understanding and better way.

**Scheme 1:**
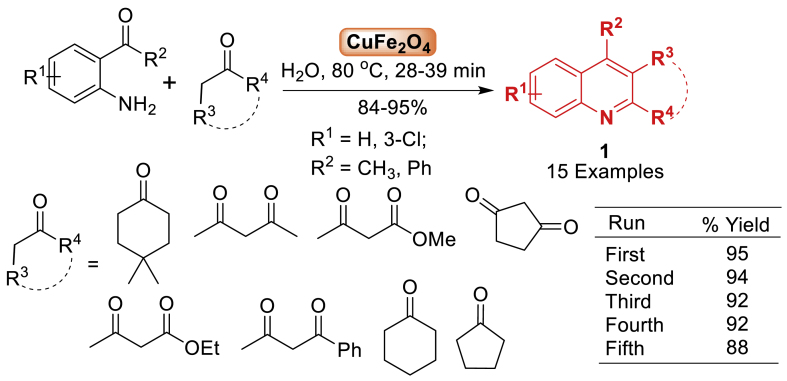
Synthesis of quinoline derivatives catalysed by CuFe_2_O_4_ NPs.

In 2014, quinoline derivatives **1** synthesized utilizing CuFe_2_O_4_ nanoparticles in aqueous medium by Farhang et al. [46] as mentioned in Scheme 1. The reaction involved the combination of substituted anilines and ketone in the presence of water as a green catalyst to afford the quinoline derivatives **1**. The protocol well tolerated with variety of starting substrates and provided products **1** in 84–95 % yields. The synthesis of nano-sized CuFe_2_O_4_ included the thermal breakdown of Cu(NO_3_)_2_ and Fe(NO_3_)_3_ in an aqueous solution with sodium hydroxide. The resulting product was verified using X-ray diffraction patterns (XRD), scanning electron microscopy pictures (SEM), and transmission electron microscopy (TEM). The nanocatalyst was reused up to five times without any significant diminishing the yield of the product as displayed in Scheme 1.

**Scheme 2:**
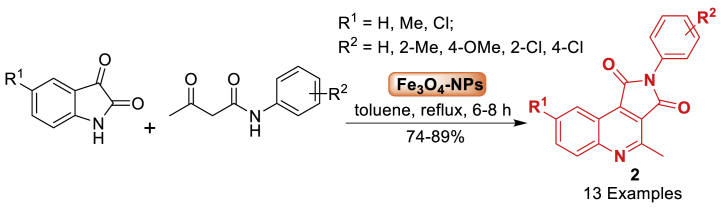
One-pot synthesis of pyrrolo[3,4-*c*]quinoline-1,3-diones using Fe_3_O_4_ nanoparticles.

In the same year, the research group of Lee [47] described the efficient formation of Fe_3_O_4_ nanoparticles through the ultrasonic treatment of Fe_2_O_3_ solution and Perilla frutescens leaf extract, which served as a dual role of reducing and capping agent. The isatin and diketoester were assembled in the presence of toluene to afford the quinoline fused derivatives **2**. All the synthesized compounds **2** were achieved in 74–89 % yields within 6–8 h of reaction time. The Fe_3_O_4_ nanoparticles shown robust catalytic activity towards pyrrolo[3,4-*c*]quinoline-1,3-dione derivatives **2** due to their facile retrieval using an external magnetic field. The results indicate that the Fe_3_O_4_ nanoparticles can serve as a catalyst in organic synthesis and can be reused up to five times without a notable decrease in their effectiveness (Scheme 2).

**Scheme 3:**
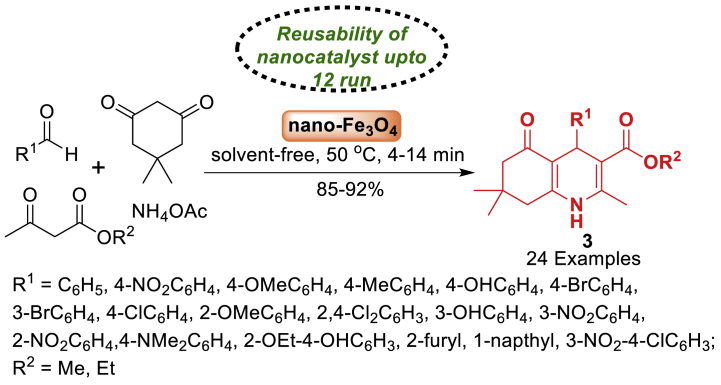
Four-component reaction of hexahydroquinolines by using nano-Fe_3_O_4_.

This study utilized magnetite nano-Fe_3_O_4_ as a green, efficient, heterogeneous, and reusable catalytic system for the atom economy synthesis of hexahydroquinolines **3** by Khakyzadeh and co-workers [48]. The synthesis involved a one-pot multi-component reaction of aryl aldehydes, dimedone, *β*-ketoesters, and ammonium acetate at 50 °C under mild and solvent-free conditions. The synthesized products **3** were obtained in 85–92 % yield within a short span of time. The electron donating and electron withdrawing groups decorated starting substrates were tolerated well towards the formation of quinoline derivatives **3**. Notably, the response surface method, namely Central Composite Design (CCD), was utilized to optimize the reaction conditions (Scheme 3).

**Scheme 4:**
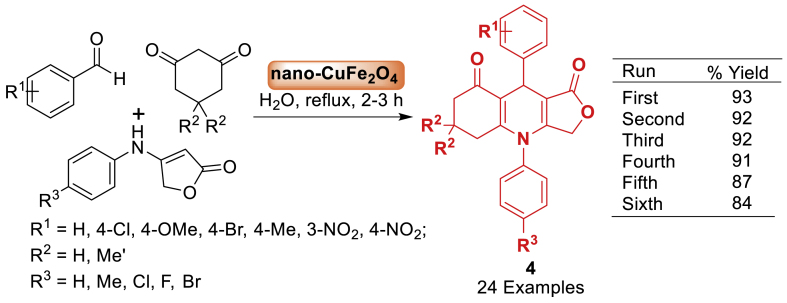
Preparation of tetrahydrofuro[3,4-*b*]quinoline-1,8(3*H*, 4*H*)-diones.

Here, an easy and effective method has been described for affording tetrahydrofuro[3,4-*b*]quinoline-1,8(3*H*,4*H*)-diones **4** through the condensation of benzaldehydes, 1,3-cyclohexanediones, and anilinolactones by Naeimi et al. [49] as displayed in Scheme 4. The current methodology was accomplished by using CuFe_2_O_4_ as a reusable nanocatalyst with strong catalytic capabilities in water. It is pleasing to note that this strategy offers significant benefits such as high yields, easy workup procedure, and minimal environment waste. Moreover, the current reaction is also applicable to a wide variety of substrate.

**Scheme 5:**
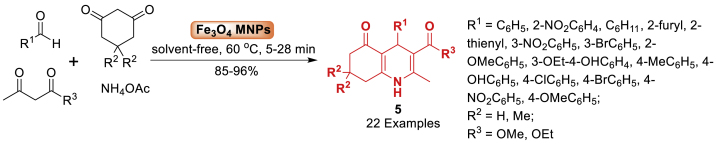
Synthesis of polyhydroquinolines in the presence of Fe_3_O_4_ NPs.

In the following year, Nasrabadi et al. [50] developed a sustainable method for the quick and effective synthesis of biologically active polyhydroquinoline derivatives **5**. The four-component reaction of benzaldehyde, *β*-ketoester, dimedone and ammonium acetate afforded quinoline framework **5** in 85–86 % yield within 5–28 min. This process utilized magnetic Fe_3_O_4_ nanoparticles (Fe_3_O_4_ MNPs) as a reusable catalyst in a solvent-free environment. Moreover, this approach has numerous advantages such as short reaction time, low catalyst loading, high yields (85–96 %), broad substrate scope, facile magnetic separation, and the ability to reuse the catalyst, when compared to traditional processes (Scheme 5).

**Scheme 6:**
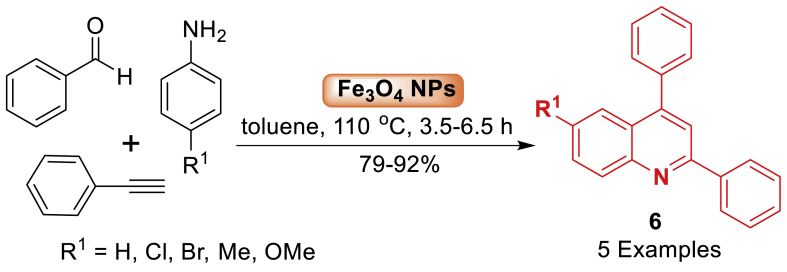
Catalytic action of Fe_3_O_4_ NPs.

Thereafter in 2015, Bhalla and co-workers [51] described a catalytic system for the synthesis of substituted quinoline derivatives **6** as illustrated in Scheme 6. Fe_3_O_4_-nanoparticles demonstrate outstanding catalytic performance in A^3^-coupling reactions to afford propargylamines, as well as in tandem intramolecular cyclization reactions for synthesizing quinolines **6** through C-H activation. Additionally, the products **6** were achieved in good to excellent yield (79–92 %) at 110 ^o^C reaction temperature. The current protocol also compatible with electron donating and electron withdrawing groups.

**Scheme 7:**
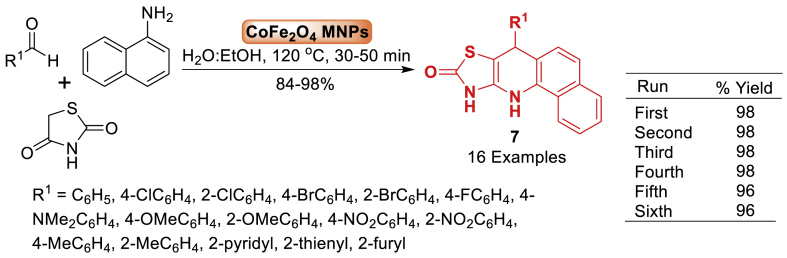
Three-component synthesis of tetrahydrobenzo[*h*] [1,3]-thiazolo[4,5-*b*]quinolin-9-ones.

The research group of Hamad [52] in 2015 first synthesized CoFe_2_O_4_ magnetic nanoparticles by a sonochemical and co-precipitation method in an aqueous solution without the use of surfactants or organic capping agents. The nanoparticles create stable dispersions in water or alcohol. Further, this magnetic nanoparticles (MNPs) were proven to be an effective and environment-friendly nanocatalyst for the one-pot multicomponent synthesis of tetrahydrobenzo[*h*] [1,3]-thiazolo[4,5-*b*]quinolin-9-one **7**. It is interesting to note that the generated compounds **7** can be easily ioslated and purified using non-chromatographic techniques. In addition to this, a high yield (84–98 %) of the products was achieved in the presence of 6 mol% of the catalyst (Scheme 7).

**Scheme 8:**
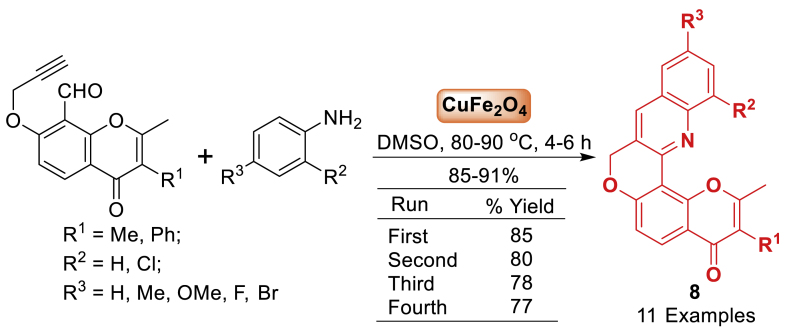
CuFe_2_O_4_ NPs-catalysed synthesis of pyrano[2’,3’:5,6]chromeno[4,3-*b*]quinolin-4-one derivatives.

In the same year, novel pyrano[2’,3’:5,6]-chromeno[4,3-*b*]quinolin-4-ones **8** have been developed using intramolecular cyclization by Babu and co-workers [53]. The Diels-Alder reaction involves the azadienes formed in the reaction between aryl amines and 8-formyl-7-(prop-2-ynyl)2,3-disubstituted chromones. This reaction is catalysed by CuFe_2_O_4_ nanoparticles in DMSO at a temperature range of 80–90 °C, resulting in high yields (85–91 %) of the desired product **8**. Furthermore, this method is particularly advantageous due to its cost-effective and reusable catalyst, high yields and straightforward nature. The structures were determined using spectroscopic data and later validated using X-ray diffraction analysis of one of the products (Scheme 8).

**Scheme 9:**
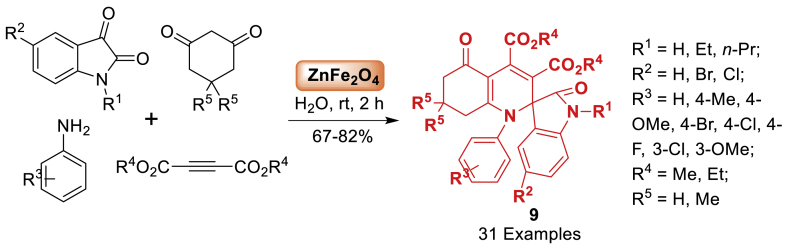
ZnFe_2_O_4_-catalysed synthesis of spiro[indoline-3,2′-quinolines].

**Scheme 10:**
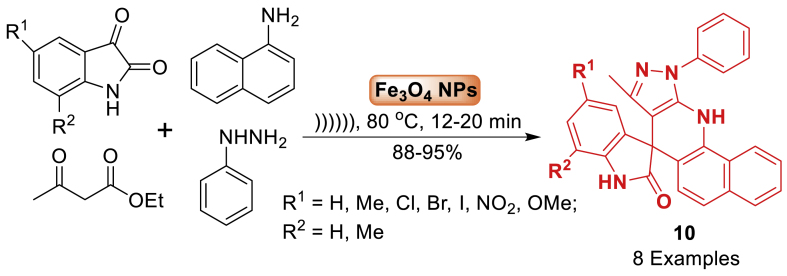
Ultrasonic-promoted synthesis of hybrids of spirooxindole-pyrazole and quinoline.

Pramanik et al. [54] first developed a bimetallic ZnFe_2_O_4_ nanopowder, acting as a dual Lewis acid-base catalyst. This system effectively catalyzes a four-component reaction to synthesize functionalized tetrahydrospiro[indoline-3,2΄-quinoline] derivatives **9** from specific starting substrates in a water medium at room temperature. The starting reactants such as isatin, aniline, alkyne and dimedone afforded targeted compounds **9** in 67–82 % yield under the green solvent. Pleasingly, the current approach is cost-effective, environmentally friendly, and sustainable due to its reduced reaction time, operational simplicity, elimination of harmful solvents, and easy recoverability and reusability of the nano catalyst. Total 31 examples were prepared by using electron donating and electron withdrawing decorated starting substrates (Scheme 9) Scheme 10.

Next, in 2016, a novel approach has been developed by Potlia et al. [55] using magnetic nanoparticles Fe_3_O_4_ as a catalyst and ultrasonic waves for the synthesis of hybrids comprising pyrazol-quinoline-oxindoles **10**, which are medicinally significant. The reaction involved ethylacetoacetate, phenylhydrazine, substituted isatin, naphthylamine at 80 ^o^C under sonication to afford the hybrids containing pyrazolo-quinoline-oxindoles **10** in 88–95 % yield. Moreover, the catalyst's recyclability and ease of recovery, the efficiency of sonication, the cleanliness of the process, the simple work-up technique, and the solvent-free approach all enhanced the usability and effectiveness of the methodology (Scheme 10).

**Scheme 11:**
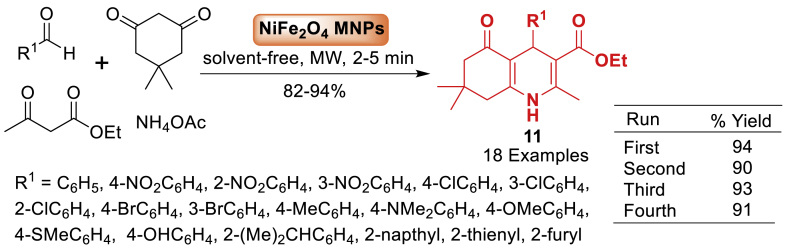
NiFe_2_O_4_-catalysed the Hantzsch condensation of polyhydroqunoline derivatives.

Under this study in the same year, a simple and efficient technique has been described by Joo et al. [56] for synthesizing polyhydroquinoline derivatives **11** by utilizing microwave radiation and magnetic nickel ferrite nanoparticles (NiFe_2_O_4_ MNPs) as a catalyst as illustrated in Scheme 11. This technique offers excellent yields (82–94 %), easy purification through recrystallization resulting in high product purity, broad substrate scope (18 examples) and quick reaction durations (2–5 min). The catalyst can be magnetically removed multiple times for reuse without a substantial decrease in the activity.

**Scheme 12:**
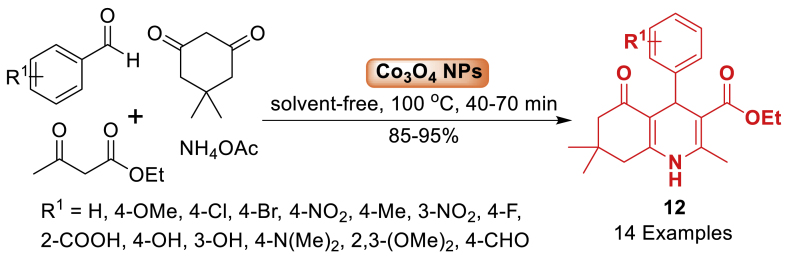
Co_3_O_4_-catalysed synthesis of polyhydroquinolines.

In 2017, the study by Elyasi and co-workers [57] presented a sustainable and effective approach for synthesizing biologically active polyhydroquinoline **12** in 85–95 % yield. The current protocol was completed by using a one-pot three-component reaction involving aromatic aldehydes, dimedone, and malononitrile in the presence of cobalt oxide nanoparticles. In this context, Co_3_O_4_ nanoparticles served as a proficient catalyst in producing polyhydroquinoline derivatives **12**. This approach has several benefits such as high efficiency, high diversity (14 examples), solvent-free reaction conditions and the possibility to reuse the catalyst (Scheme 12).

**Scheme 13:**
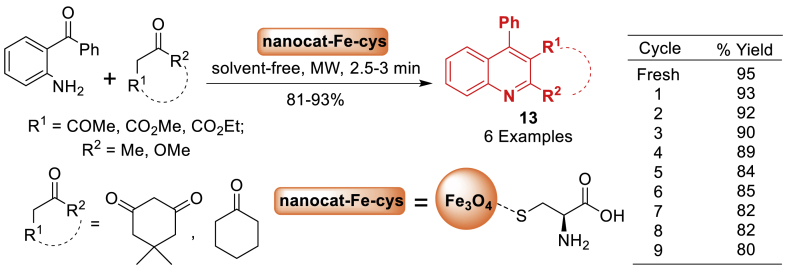
Friedlander method catalysed by Fe_3_O_4_-Cys MNPs.

Across the globe, nano magnetite supported catalysis is an important and expanding area in catalytic research that is utilized in organic synthesis. In 2018, the research work by Shelke et al. [58] focused on developing a nano-magnetite supported cysteine organocatalysts. Thereafter it used in catalysis and synthetic organic chemistry to afford the quinoline derivatives **13** by the reaction of substituted aniline and carbonyl compounds under solvent free reaction conditions. Additionally, the current strategy has several additional advantages such as nano catalyst, solvent free reaction, microwave condition, short reaction time (2.5–3 min) and high yield of the products **13** (Scheme 13).

**Scheme 14:**
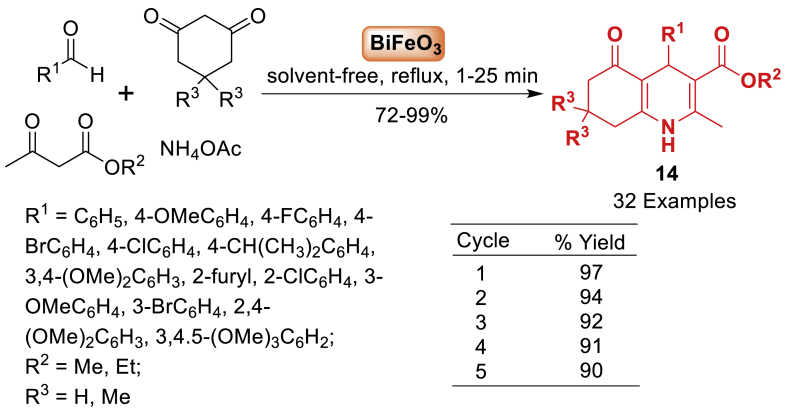
Formation of various polyhydroquinolines with substituted aromatic aldehydes.

In the following year, the research group of Rajput [59] developed a novel sucrose chelated Bismuth ferrite (BiFeO_3_) nanoparticles catalyst using an auto combustion method. Various calcination temperatures ranging from 150 to 850 °C were used to fabricate homogeneous BiFeO_3_ nanoparticles. Furthermore, the BiFeO_3_ nanoparticles were used as a catalyst for generating polyhydroquinoline derivatives **14**. A one-pot four-component cyclization reaction involved benzaldehyde, ethylacetoacetate/methylacetoacetate, dimedone/cyclohexane-1,3-dione, and ammonium acetate to synthesize polyhydroquinoline derivatives **14**. This was achieved without the use of a solvent under refluxing conditions, resulting in high product yields (72–99 %). It is interesting to note that electron donating and electron withdrawing groups played very well to achieve the quinoline frameworks **14**. The BiFeO_3_ nanocatalyst demonstrates efficient magnetic separation, recyclability, and reusability without any functionalization or surface coatings (Scheme 14).

**Scheme 15:**
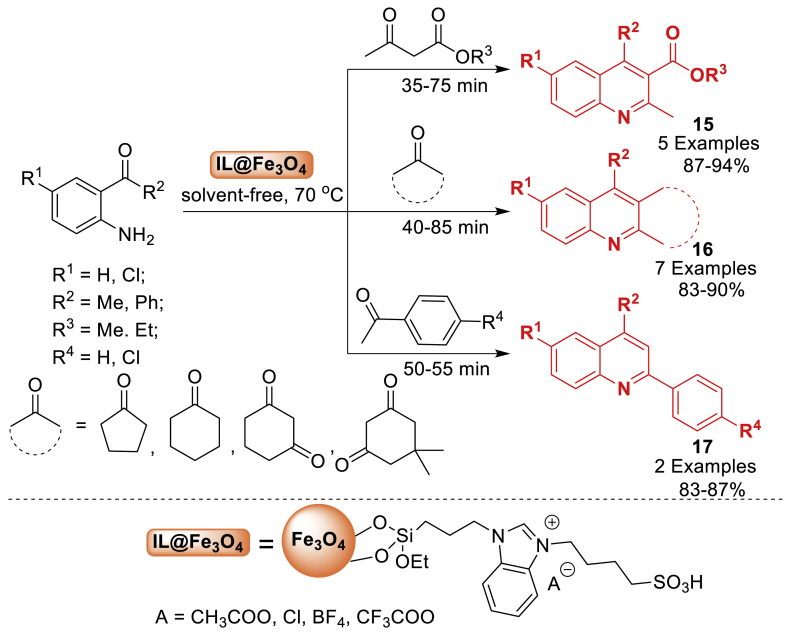
Friedlander reaction for the synthesis of quinoline and fused polycyclic quinolines.

Further, in 2019, Fe_3_O_4_ nanoparticles were functionalized to construct catalysts consisting of magnetite nanoparticles supported by Dadhania and co-workers [60]. The synthesized catalysts were tested for their catalytic effectiveness by using them to generate biologically active substituted quinoline and fused polycyclic quinoline derivatives **15–17**. The IL-IL@Fe_3_O_4_ catalyst with acetate ion showed promise in catalysing the Friedlander reaction, resulting in high yields (83–94 %) of the final products **15–17** under green reaction conditions. The procedure became environment-friendly due to the easy separation of the catalyst using an external magnet and its capacity to be reused for up to six reaction cycles (Scheme 15).

**Scheme 16:**
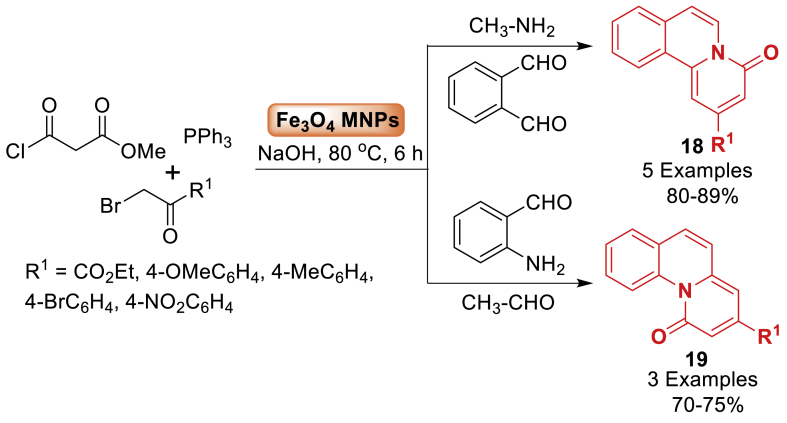
Multicomponent reaction for the synthesis of pyrido[2,1‐*a*] isoquinoline and pyrido[1,2‐*a*]quinoline derivatives.

In the same year, the study by Noushin’s group [61] examined the efficient production of pyrido[2,1‐*a*]isoquinolines **19** and pyrido[1,2‐*a*]quinolines **18** through a multicomponent reaction involving phthalaldehyde, methyl amine, methyl malonyl chloride, alkyl bromides, and triphenylphosphine. This reaction was catalysed by a small quantity of Fe_3_O_4_‐MNPs in the presence of aqueous sodium hydroxide at 80 °C, resulting in high yields (70–89 %). Moreover, antioxidant activity of newly synthesized compounds **18**, **19** was investigated utilizing DPPH radical trapping and ferric ion reduction studies, and results were compared with synthetic antioxidants *tert*-butyl-hydroquinone (TBHQ) and butylated hydroxytoluene (BHT) (Scheme 16).

**Scheme 17:**
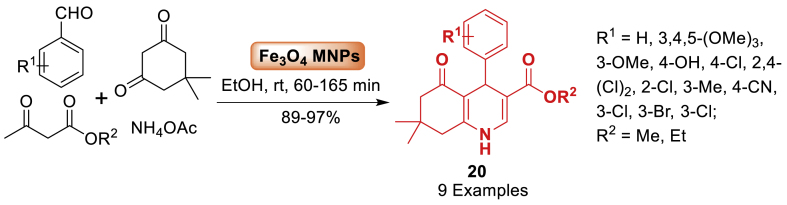
One-pot formation of polyhydroquinoline derivatives.

A unique magnetic chitosan-terephthaloyl-creatine bionanocomposite was fabricated for the first time by Maleki et al. [62] in 2019. Chitosan bio polymeric chains were functionalized with synthesized creatine-terephthaloyl chloride ligands. The functionalized polymeric substrate was magnetized by *in-situ* preparation of Fe_3_O_4_ magnetic nanoparticles. The catalytic efficiency and performance of the new magnetic bionanocomposite were assessed in Hantzsch condensation reactions, in addition to identifying its distinctive properties (Scheme 17). Additionally, it can be effectively used to produce polyhydroquinoline **20** derivatives in high yields (89–97 %) and short reaction times, following green chemistry principles.

**Scheme 18:**
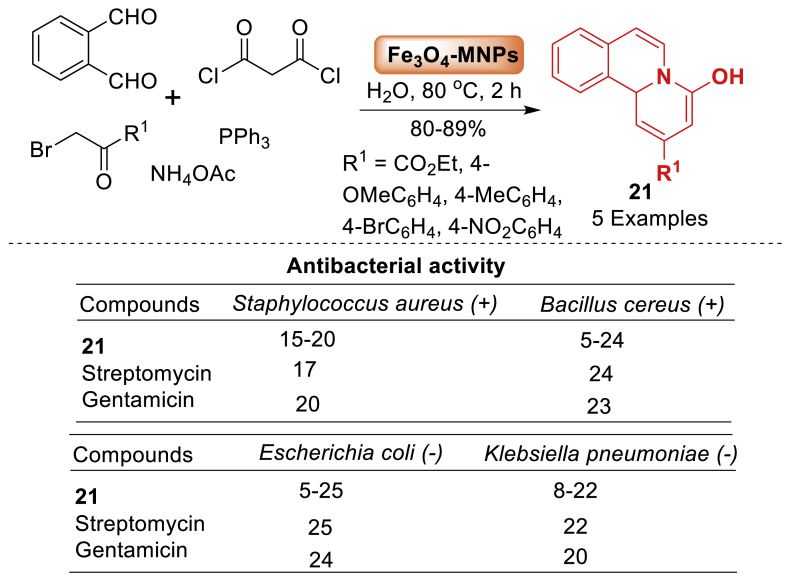
One-pot three-component method for the synthesis of pyrido[2,1-*a*]isoquinolines.

Here, the multicomponent reactions involved isoquinoline, methyl malonyl chloride, alkyl bromides, and triphenylphosphine in the presence of catalytic quantity of Fe_3_O_4_-MNPs, the synthesis of pyrido[2,1-*a*]isoquinolines **21** in excellent yield were explored by Shafaei et al. [63] (2022) as depicted in Scheme 18. Additionally, utilizing the DPPH radical trapping and reduction of ferric ion tests, antioxidant activity was investigated for a few newly synthesized compounds **21**, and findings were compared with synthetic antioxidants (TBHQ and BHT). The disk diffusion test was used to investigate the antibacterial activity of several produced compounds against both Gram-positive and Gram-negative bacteria. The disk diffusion test results demonstrated that compounds **21** inhibited the growth of bacteria.

### 2.2 Role of dopped nanoparticles in the synthesis of quinoline derivatives

This section focuses on the synthesis of quinoline through the utilization of dopped magnetic nanoparticles as a catalyst. This section is further divided into three different categories as inorganic dopped nanoparticles, organic dopped nanoparticles and hybrid nanoparticles.

#### 2.2.1 Role of inorganically dopped nanoparticles in the synthesis of quinoline derivatives

Here, we have discussed the role of inorganically dopped nanoparticles on the synthesis of quinoline tether/fused molecular hybrids. The detailed study is presented herein.

**Scheme 19:**
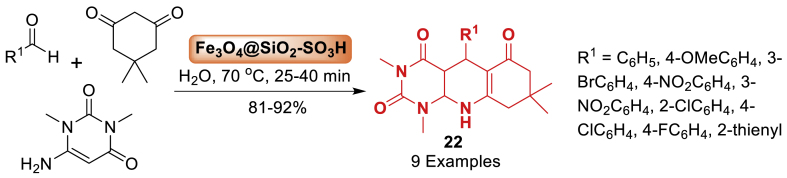
Synthesis of pyrimido[4,5-*b*]quinolines using Fe_3_O_4_@SiO_2_-SO_3_H as a nanocatalyst.

In 2013, Nemati et al. [64] synthesized a magnetic nanoparticle supported silica sulfuric acid as a catalyst. Thereafter this catalyst played a highly effective role in the synthesis of pyrimido[4,5-*b*]quinolines **22** in an aqueous environment as presented in Scheme 19. It is noted that the reaction of substituted benzaldehyde, dimedone and aniline afforded quinoline fused molecular hybrids in 81–92 % yield within 25–40 min. All the starting substrates tolerated well towards this transformation. It is interesting to note that the Fe_3_O_4_@SiO_2_-SO_3_H compound could be easily retrieved with an external magnet and reused multiple times without a notable decrease in reactivity.

**Scheme 20:**
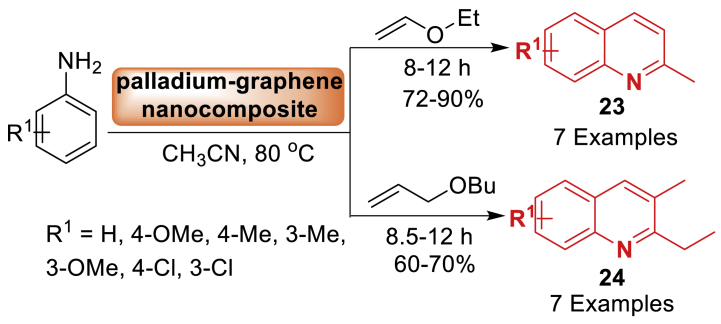
Palladium-graphene nano composite-catalysed synthesis of quinoline tethered derivatives.

Afterwards, in 2014, the research team of Jain [65] unfolded the synthesis of magnetically separable palladium-graphene nanocomposite as a fully magnetically separable catalyst. This catalyst showed its efficiency in the formation of 2-alkyl quinolines **23**–**24** through the reaction of anilines with alkenyl ethers (Scheme 20). The catalyst displayed increased catalytic efficiency due to the uniform decorating of Pd nanoparticles on the support, and it maintained its effectiveness through six repeated cycles without any alteration. Delightedly, the synthesized products **23**–**24** were obtained in 72–90 % and 60–70 % yields.

**Scheme 21:**
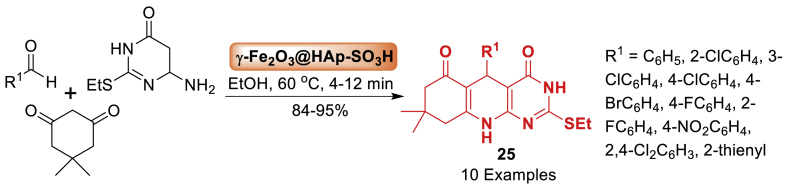
Synthesis of pyrimido[4,5-*b*]quinolines derivatives.

Next, Sabet et al. [66] developed a hydroxyapatite-encapsulated-*γ*-Fe_2_O_3_ [*γ*-Fe_2_O_3_@HAp-SO_3_H] as a magnetically recoverable nanocatalyst. Afterwards, this catalyst utilized in the one-pot synthesis of novel pyrimido[4,5-*b*]quinolines derivatives **25** in a three-component strategy as mentioned in Scheme 21. The reaction involved benzaldehyde, dimedone and substituted 2-aminopyrimidine under 60 ^o^C within 4–12 min. Total 10 examples were obtained in 84–95 % yield. The procedure offered an efficient, environmentally friendly technique for producing the final products **25** with high yields (84–95 %).

**Scheme 22:**
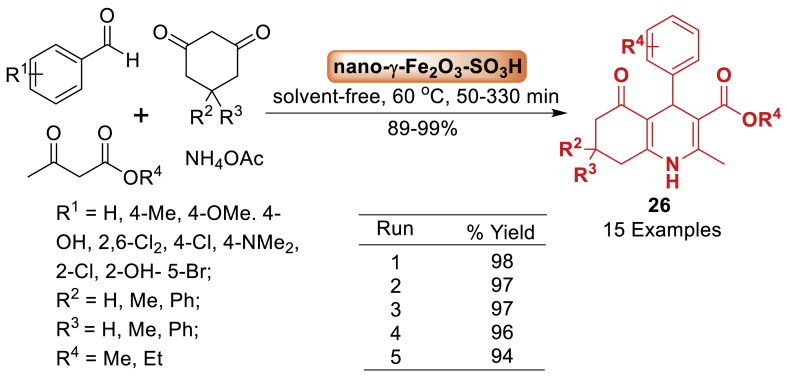
Solvent-free synthesis of polyhydroquinolines.

A straightforward and effective method was developed by Azhari and co-workers [67] for generating polyhydroquinolines **26**. It includes a one-step process called Hantzsch condensation, which involves aromatic aldehydes, 1,3-cyclohexane diones, alkyl acetoacetate, and ammonium acetate with a small amount of nanomagnetic-supported sulfonic acid, without using any solvent (Scheme 22). The approach provides numerous benefits such as high yields, broad substrate scope, solvent free reaction condition, short reaction times, an uncomplicated work-up process, and the possibility to reuse the catalyst for multiple times. The starting substrates decorated with electron donating and electron withdrawing afforded the quinoline tethered derivatives **26** without compromising the optimal reaction condition.

**Scheme 23:**
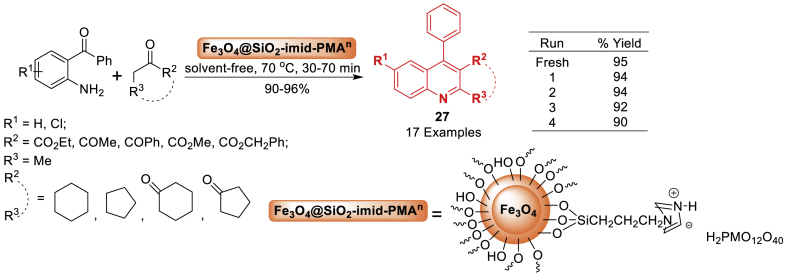
Synthesis of quinolines under solvent-free conditions.

Here, Javidi’s research team [68] reported a simple one-step method for developing poly-substituted quinoline **27** derivatives. The process involved 2-aminobenzophenones and ethylacetoacetate or ketones with Fe_3_O_4_@SiO_2_-imid-PMA^n^ nanoparticles as environment-friendly and recyclable catalysts in a solvent-free environment. The reaction occurs efficiently, resulting in high yields and exceptional purity. The nanocatalyst can be reused up to four times without a significant decrease in catalytic activity. The products **27** were obtained in the range of 90–96 % yields (Scheme 23).

**Scheme 24:**
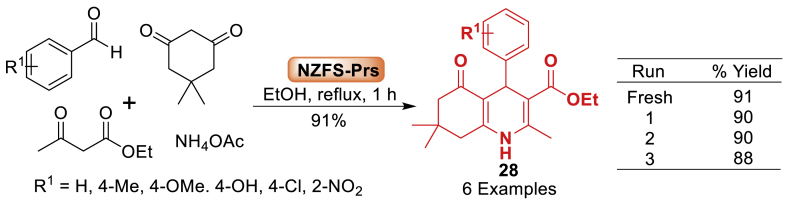
Four-component synthesis of polyhydroquinolines using NZFS‐Prs as a nano catalyst.

Under this study, group of Laali [69] disclosed a novel magnetic acidic catalyst supporting Preyssler (H_14_[NaP_5_W_30_O_110_]) heteropoly acid on silica-coated nickel zinc ferrite nanoparticles (Ni_0.5_Zn_0.5_Fe_2_O_4_@SiO_2_). The catalytic activity was studied for synthesizing polyhydroquinoline compounds **28** using the Hantzsch process (Scheme 24). The Hantzsch reaction involved aromatic aldehyde, diketoester, dimedone and ammonium acetate at refluxing. Interestingly, the protocol well tolerated with a wide variety of substrates. It is interesting to note that the catalyst expedited the reactions, resulting in excellent yields in less than 1 h.

**Scheme 25:**
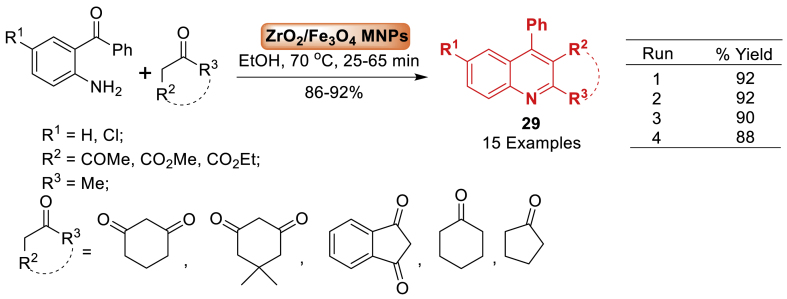
Synthesis of ethyl 2-methyl-4-phenylquinoline-3-carboxylate catalysed by ZrO_2_/Fe_3_O_4_-MNPs.

The study by Shirini et al. [70] developed a practically suitable, and environment-friendly approach for synthesizing quinoline derivatives **29** by a Friedlander reaction employing ZrO_2_/Fe_3_O_4_-MNPs as an efficient and reusable catalyst (Scheme 25). The ZrO_2_/Fe_3_O_4_-MNPs morphology was analysed using XRD, FT-IR, SEM, TEM, and VSM techniques. The advantages of this approach include easy reaction conditions, simple and efficient reaction processes, high product yields (86–92 %), and favourable reaction durations. The catalyst can be retrieved with an external magnetic field and reused up to three times without a significant decline in its catalytic efficiency. The total 15 examples were achieved in 86–92 % yield.

**Scheme 26:**
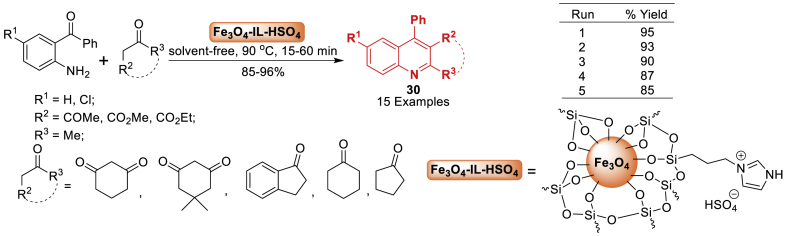
Preparation of quinoline tethered derivatives catalysed by Fe_3_O_4_-IL-HSO_4_.

In the following year, a method for preparing Fe_3_O_4_ nanoparticles and a supported Brønsted acidic ionic liquid, 1-methyl-3-(3-trimethoxysilylpropyl)imidazolium hydrogen sulfate (Fe_3_O_4_-IL-HSO_4_), has been outlined by Abbaspour and co-workers [71]. This combination serves as an effective magnetic catalyst for synthesizing polysubstituted quinolines **30** *via* Friedlander condensation of 2-aminoaryl ketones with 1,3-dicarbonyl compounds under solvent-free conditions. This technology stands out for its environmental friendliness, ease of operation, high yield (85–96 %) in a short reaction time (15–60 min), simple product isolation, and the catalyst's potential for exceptional reusability (Scheme 26).

**Scheme 27:**
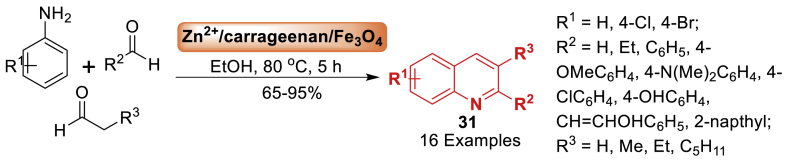
One‐pot three‐component formation of quinoline derivatives.

Afterwards, in 2017, Tavakol et al. [72] documented the initial presence of zinc cation on ƛ‐carrageenan/Fe_3_O_4_ magnetic nanoparticles. This catalytic system, which is both green and efficient, was utilized in the formation of biologically active quinolines **31**. The products **31** were acquired with good to excellent yields (52–95 %) by a one-step reaction process (Scheme 27). The approach has numerous benefits, including gentle reaction conditions, high diversity, simple work-up, utilization of a reusable magnetic catalyst, and excellent product yields.

**Scheme 28:**
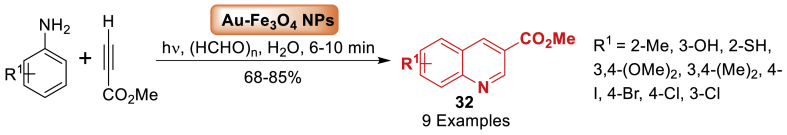
One-pot approach for the synthesis of derivative of quinolines.

Next, in 2017, a AuFe_3_O_4_ nano hybrid materials has been synthesized by the team of Bhalla [73] as illustrated in Scheme 28. Furthermore, AuFe_3_O_4_ nano hybrid materials were used as a highly effective and reusable catalytic system for activating C(sp^2^)H bonds in electron-rich anilines to achieve versatile quinoline carboxylates **32** through C-H activation, carbonylation, and annulation in mild and nature friendly conditions (such as aqueous media, room temperature, visible light exposure, and aerial conditions). The reaction led to final products **32** in 68–85 % yields within 6–10 min.

**Scheme 29:**
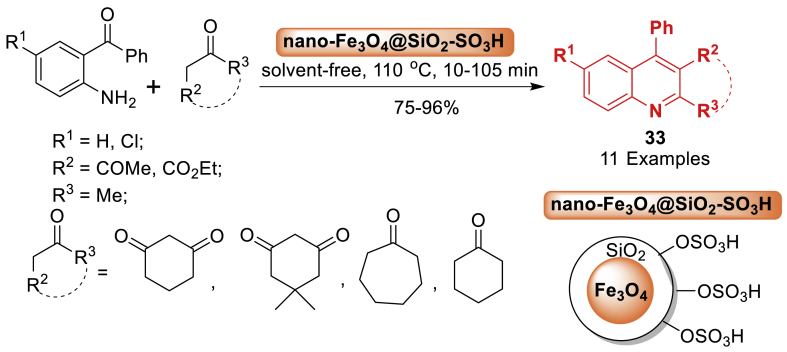
Synthesis of substituted quinolines catalysed by Fe_3_O_4_@SiO_2_-SO_3_H

A new approach has been devised by Fallah-Mehrjardi et al. [74] for the Friedlander synthesis of substituted quinolines **33** as summarized in Scheme 29. It involved a condensation reaction between 2-aminoaryl ketones and *α*-methylene ketones using a catalytic quantity of nano Fe_3_O_4_@SiO_2_-SO_3_H at 110 °C without solvent. The reactions are easily completed, yielding compounds **33** in good to excellent yield (75–96 %). The results showed multiple benefits of method, such as high product yields, broad substrate scope (11 examples), easy work-up process, straightforward operation, nature friendly reaction conditions, catalyst reusability, and sustainability by eliminating toxic catalysts and solvents.

**Scheme 30:**
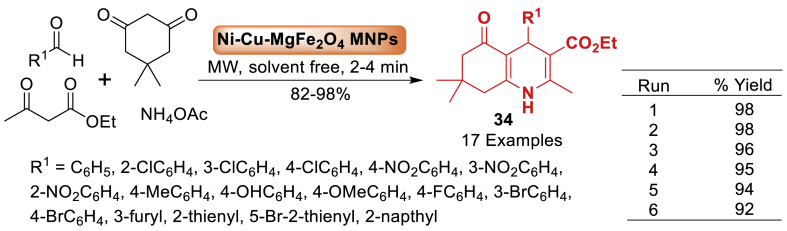
MNPs-catalysed Hantzsch synthesis of polyhydroqunoline derivatives.

Researchers are interested in utilizing natural gel for the cost-effective, non-toxic, economically viable, and eco-friendly fabrication of nanoparticles (Scheme 30). In this direction, Moradi’s group [75] synthesized Ni_0.35_Cu_0.25_Mg_0.4_Fe_2_O_4_ MNPs using tragacanth gum as a biotemplate and metal nitrates as the metal supply by the sol-gel process, without the use of any organic compounds. Ni-Cu-Mg ferrite nanoparticles were utilized as a catalyst for achieving polyhydroquinoline derivatives **34** by multi-component reactions under microwave irradiation. The approach is characterized by a straightforward procedure, broad substrate scope, gentle reaction parameters, brief reaction durations (2–4 min), utilization of a cost-effective catalyst, and high product **34** yields ranging from 82 to 98 %.

**Scheme 31:**
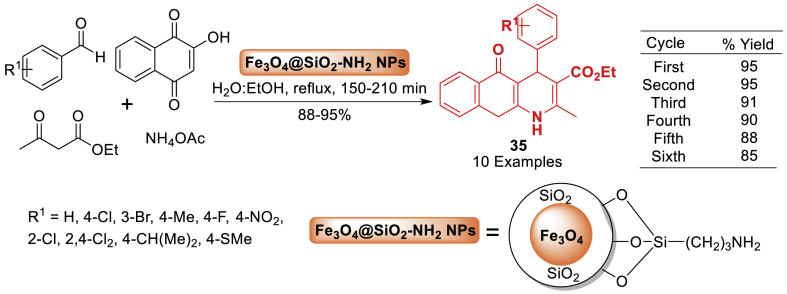
Formation of tetrahydrobenzo[*g*]quinoline-5,10-diones.

Under this study in 2017, Eshkevari et al. [76] presented a sustainable method for synthesizing biologically active heterocyclic compounds **35**, such as 2-amino-5,10-dihydro-5,10-dioxo-4*H* benzo[*g*] as displayed in Scheme 31. Synthesis of tetrahydrobenzo[*g*]quinoline-5,10-diones **35** using one-pot multi-component reactions with the assistance of Fe_3_O_4_@SiO_2_-NH_2_ nanocomposite. This protocol describes the synthesis and application of amino-functionalized Fe_3_O_4_@SiO_2_ as an efficient and recyclable nanocatalyst. In addition to this work, this approach has benefits such as high yields (88–95 %), high diversity (10 examples), easy work-up, and the opportunity to reuse the catalyst.

**Scheme 32:**
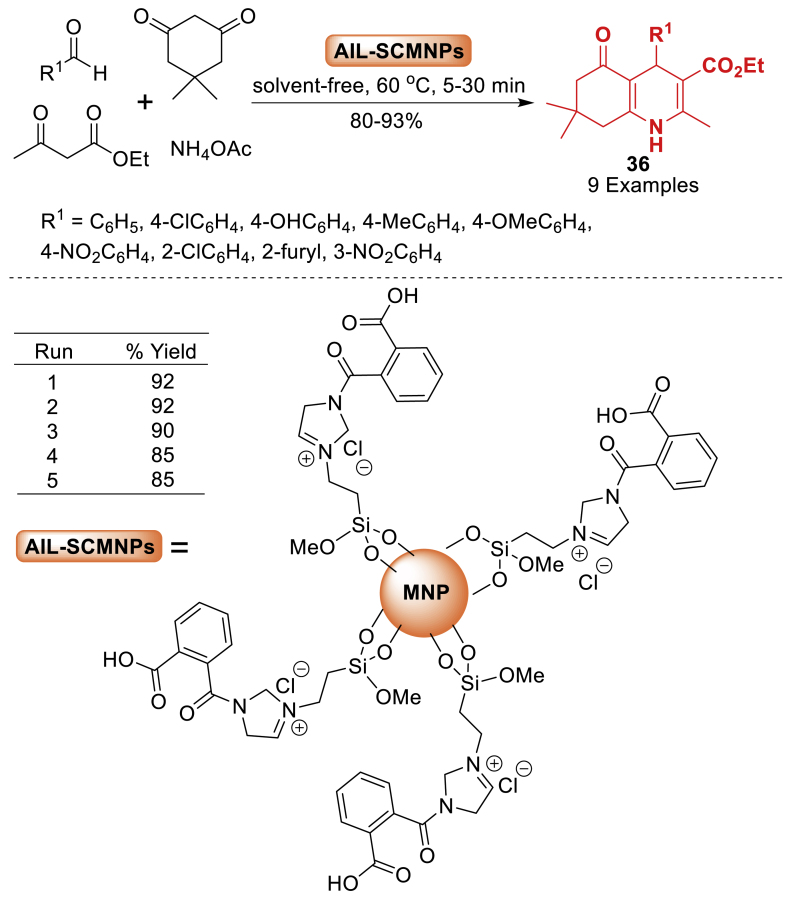
Mild and solvent-free synthesis of polyhydroquinoline derivatives.

Here, the research group of Heidarizadeh [77] synthesized polyhydroquinoline derivatives **36** using a magnetic catalyst composed of novel acidic ionic liquid functionalized silica modified Fe_3_O_4_ nanoparticles as presented in Scheme 32. This was achieved under solvent-free conditions through a four-component combination of *β*-ketoester, aldehydes, and ammonium acetate. This technology offers advantages such as a straightforward procedure, short reaction time (5–30 min), and high efficiency. Interestingly, the catalyst showed good thermal stability and could be recycled effectively.

**Scheme 33:**
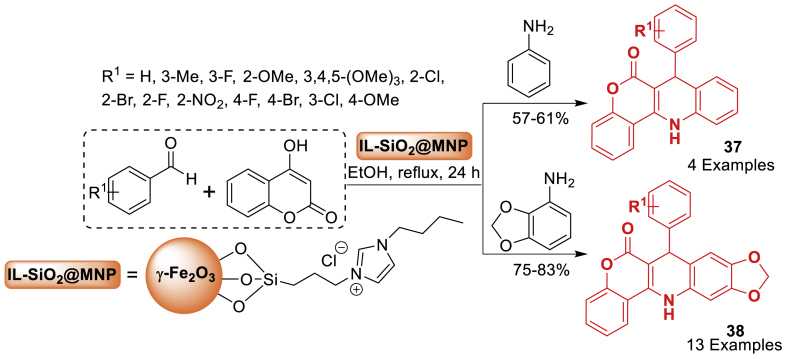
Synthesis of 6*H*-chromeno[4,3-*b*]quinolone derivatives.

In this work, Mahdavi et al. [78] unfolded imidazole ionic liquid (IL)-functionalized silica@γ-Fe_2_O_3_ (IL-SiO_2_@MNP) as depicted in Scheme 33. The catalyst is identified by transmission electron microscopy, scanning electron microscope, vibrating sample magnetometer, dynamic light scattering, and Fourier transform infrared spectroscopy. IL-SiO_2_@MNP shown high efficiency in catalysing the production of 6*H*-chromeno[4,3-*b*]quinolin-6-one derivatives **37**–**38** through a multicomponent reaction including 4-hydroxycoumarin, anilines, and benzaldehydes. The nanocatalyst is both magnetically separable and easily be recovered, demonstrating effective activity for up to 10 runs.

**Scheme 34:**
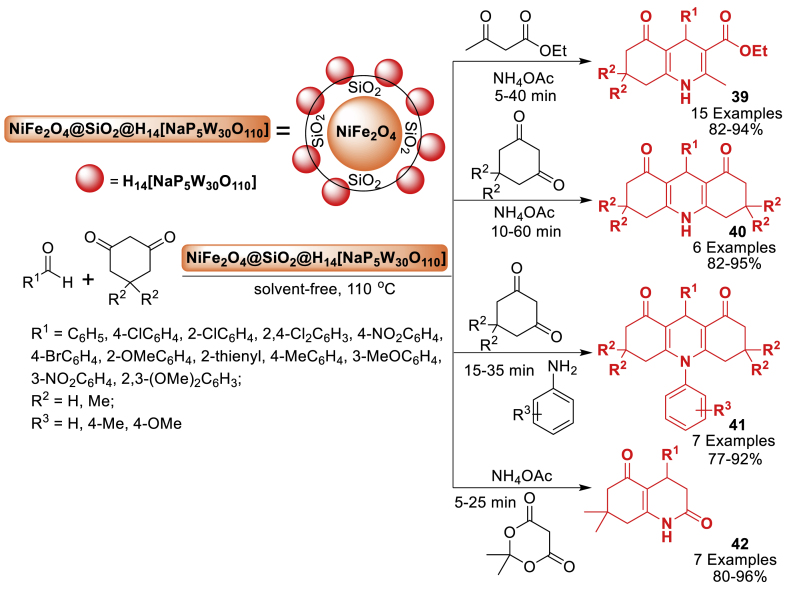
One-pot method for the synthesis of polyhydroquinolines, 1,8-dioxodecahydroacridines, and 2,5-dioxo-1,2,3,4,5,6,7,8-octahydroquinolines.

A versatile and highly efficient heterogeneous catalyst, NiFe_2_O_4_@SiO_2_-H_14_[NaP_5_W_30_O_110_], was successfully synthesized by the research group of Seresht [79] for the one-pot multicomponent synthesis of 1,4-dihydropyridine **39**–**42** derivatives under solvent-free conditions (Scheme 34). The reaction of aromatic aldehyde and dimedone afforded the wide variety of final products **39**–**42** in 77–96 % yield. The protocol has several advantages such as solvent free conditions, broad substrate scope, reusability and easy separation etc. The catalyst can be retrieved using a magnet and reused up to four times without a noticeable decrease in efficiency. This protocol provided a wide variety of products **39**–**42** due to the various type of starting substrates having electron donating and electron withdrawing groups.

**Scheme 35:**
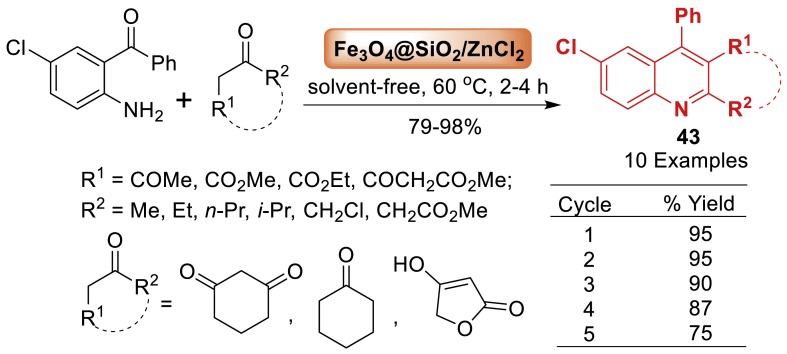
Friedländer synthesis of quinoline derivatives through Fe_3_O_4_@SiO_2_/ZnCl_2_

Sepahvand’s group [80] developed a zinc chloride supported silica-coated magnetic nanoparticles of Fe_3_O_4_ to create a magnetic nanocatalyst named Fe_3_O_4_@SiO_2_/ZnCl_2_ (Scheme 35). This catalyst was utilized to synthesize quinolines **43** by the Friedlander synthesis method using 2‐aminoaryl ketones and *α*‐methylene ketones. It is interesting to note that the products **43** were achieved in 79–98 % yields under solvent-free reaction conditions. Total 10 examples were prepared under this study. Fe_3_O_4_@SiO_2_/ZnCl_2_ exhibited superior catalytic activity compared to ZnCl_2_ alone and may be reused multiple times without any notable decrease in activity.

**Scheme 36:**
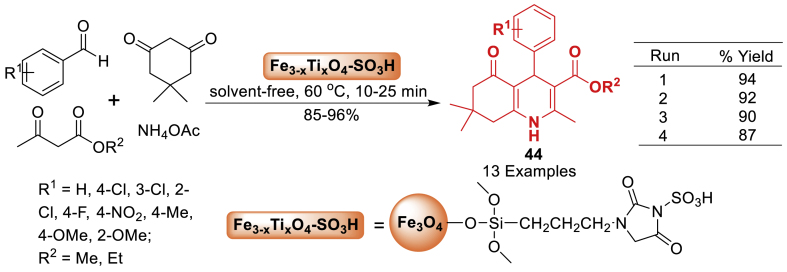
Solvent-free synthesis of hexahydroquinolines.

Thereafter in 2019, Badalkhani et al. [81] fabricated an acidic nanocatalytic system, by reacting Fe_3-x_Ti_x_O_4_ MNPs with 3-chloropropyltrimethoxysilane and imidazolidine-2,4-dione in a one-pot reaction, and then functionalizing them with chlorosulfonic acid. The catalytic potential of this nanocatalyst was assessed in a one-pot four-component condensation reaction including aromatic aldehydes, dimedone, alkyl acetoacetates, and ammonium acetate for the formation of hexahydroquinoline derivatives **44**. The final products **44** were obtained in 85–96 % yields under the solvent-free reaction conditions. The reactions progressed easily in the absence of solvent, resulting in high yields of the desired hexahydroquinolines **44**. Furthermore, the synthetic nanocatalyst showed outstanding reusability for up to four consecutive uses (Scheme 36).

**Scheme 37:**
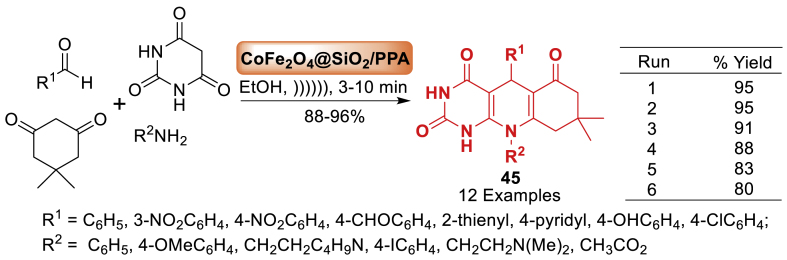
MCRs for the synthesis of dihydropyrimido[4,5‐*b*]quinolinetriones.

Next, in 2019, Mahdipour et al. [82] developed a novel and environment-friendly approach for synthesizing dihydropyrimido [4,5‐*b*]quinolinetrione derivatives **45** as mentioned in Scheme 37. A high-performance solid acid catalyst was synthesized *via* a three-step process and CoFe_2_O_4_ nanoparticles were achieved utilizing the co-precipitation method. It was then employed as a catalyst in the synthesis of dihydropyrimido[4,5‐*b*]quinolinetrione derivatives **45**. In addition to above, the approach offered various advantages such as environmentally favourable settings, quick reaction times (3–10 min), high product yields (88–96 %), and easy workup.

**Scheme 38:**
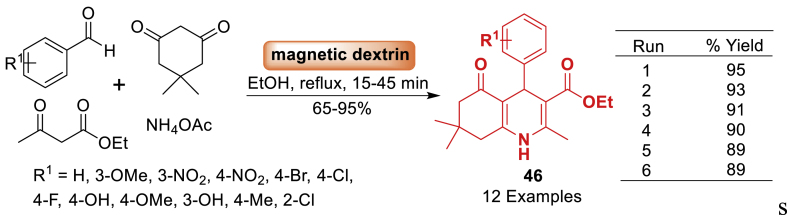
Synthesis of polyhydroquinoline derivatives.

A magnetic dextrin nanobiocomposite was generated by the research team of Esmaeili [83] using a straightforward chemical co-precipitation process (Scheme 38). To assess the catalytic efficiency of the synthesized hybrid catalyst, it was utilized in the synthesis of biologically active polyhydroquinoline derivatives **46** through a four-component condensation reaction involving aromatic aldehyde, ethyl acetoacetate, dimedone, and ammonium acetate in ethanol under reflux conditions. The current method offers several advantages, including the utilization of green and biopolymer-based catalyst, a straightforward procedure, easy reaction conditions, short reaction times (15–45 min), broad substrate scope (12 examples), excellent product yields (65–95 %), and the ability to reuse the catalyst for up to five consecutive runs without a significant decrease in catalytic performance.

**Scheme 39:**
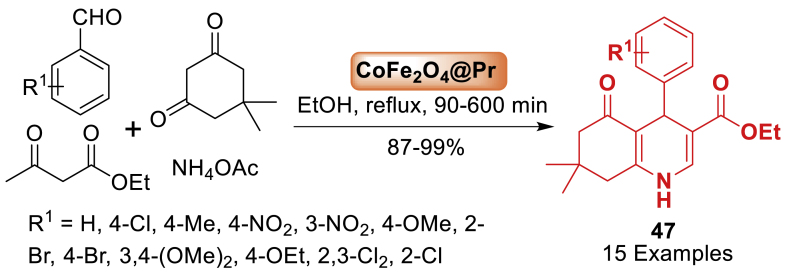
CoFe_2_O_4_@Pr-assisted synthesis of polyhydroquinoline derivatives.

Furthermore, Mohammadi and co-workers [84] in 2020 described a simple method for attaching a praseodymium (III) complex to the magnetic nanoparticles' surface using accessible materials as depicted in Scheme 39. The samples were analysed using FT-IR, TGA, SEM, XRD, and EDX techniques and utilized in the synthesis of polyhydroquinoline **47** derivatives. The reaction yields were found to be good to excellent when using the synthesized nanocatalyst. The notable aspect of this procedure is the utilization of a retrievable and novel magnetic nanocatalyst in these reactions. Total 15 examples were achieved in 87–99 % yields.

**Scheme 40:**
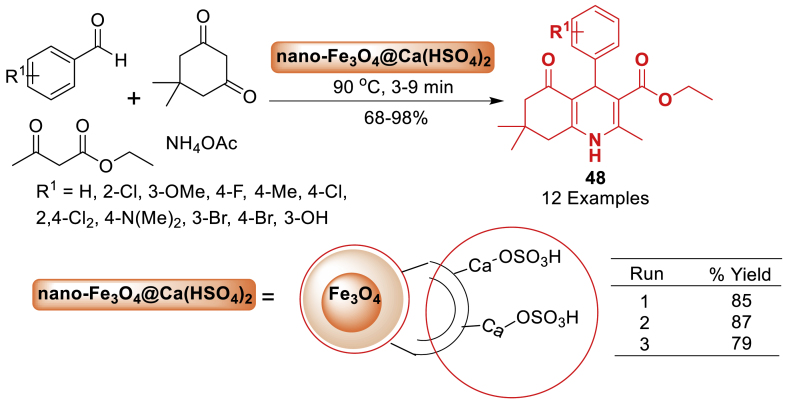
Synthesis of hexahydroquinoline derivatives.

In the same year, the research group of Gilan [85] developed a nano catalyst as Fe_3_O_4_@Ca(HSO_4_)_2_ as presented in Scheme 40. The catalytic activity of the prepared catalyst was investigated in the synthesis of hexahydroquinoline **48** derivatives. The present work's primary advantages include high yield, low reaction time, solvent-free conditions, good to excellent yield and short reaction time. Total 12 examples were prepared in 68–98 % yields within 3–9 min of reaction time.

**Scheme 41:**
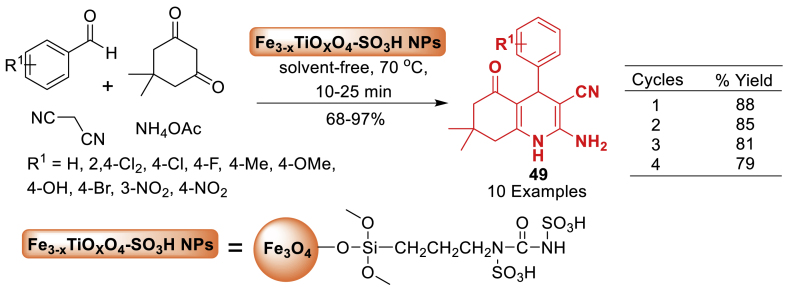
Four-component Fe_3-x_Ti_x_O_4_-SO_3_H NPs-mediated synthesis of 2-amino-3-cyano-hexahydroquinolines.

The efficacy of sulfonated-titanomagnetite nanoparticles (Fe_3-x_Ti_x_O_4_-SO_3_H NPs) as a valuable and recyclable nanocatalyst for the synthesis of hexahydroquinoline derivatives **49** *via* a multi-component reaction method in solvent-free conditions was thoroughly examined by Mona et al. [86] in 2021. The reaction of starting substrates under the solvent free conditions afforded the final products **49** in 68–97 % yields. According to the findings, the nanocatalyst used has several advantages, including high catalytic activity in a short reaction time, good-to-excellent isolated yields in most cases, an easy workup process, a smooth processing feature of the reactions, easy recovery using an external magnetic field, and reusability for four times without significant loss in activity (Scheme 41).

**Scheme 42:**
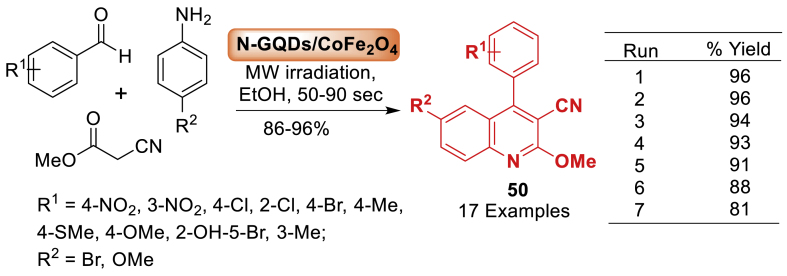
MW-assisted synthesis of quinoline-3-carbonitrile derivatives.

Under this work, Safaei-Ghomi et al. [87] developed a novel nitrogen-doped graphene quantum dots (N-GQDs). Following synthesis, the catalytic activity of the designed CoFe_2_O_4_ nanocomposites was tested, and the results showed that spherical > rod > prism > cubic. The uniform spherical morphology allows for more accessible active areas. The unique nano-sized N-GQDs/CoFe_2_O_4_ magnetic spherical composite was then easily manufactured using a green, low-cost, and simple hydrothermal method (Scheme 42). The designed composite was used as an efficient magnetic nanocatalyst for MW-assisted one-pot synthesis of quinoline 3-carbonitrile derivatives **50** (83–96 % yields) in the shortest reaction time (50–90 s).

**Scheme 43:**
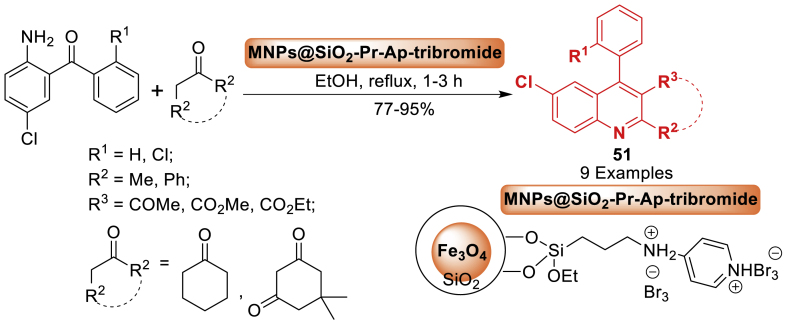
Formation of quinoline derivatives using MNPs@SiO_2_-Pr-AP-tribromide as a nano catalyst.

After that, in 2022, the research team of Alavinia [88] reported magnetic iron oxide nanoparticles functionalized with various chemical groups (Scheme 43). A novel nano-catalyst, ionic liquid nano-magnetic pyridinium-tribromide (MNPs@SiO_2_-Pr-AP-tribromide), was developed in this study. The nano catalyst effectively facilitated the production of quinoline derivatives **51** by enabling a one-pot reaction between 2-amino-5-chlorobenzophenone and pentane-2,4-dione. This catalyst has benefits such as simple preparation, extensive surface area, renewable nature, strong thermal stability, high activity, reduced reaction time, enhanced efficiency, straightforward purification, and easy reaction conditions.

**Scheme 44:**
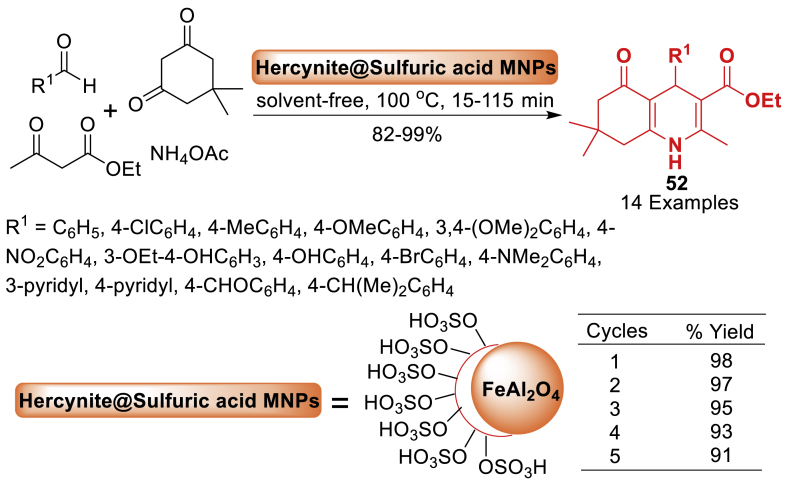
Solvent-free Hantzsch synthesis of polyhydroquinoline derivatives.

In this work, Ghorbani-Choghamarani and team [89] described the fabrication of hercynite@sulfuric acid as a new nanomagnetic solid acid catalyst. This catalyst consists of sulfuric acid catalytic sites located on the surface of hercynite MNPs, serving as the catalytic support. The synthesized nanocomposite was thoroughly analysed utilizing several physicochemical techniques such as FT-IR, XRD, EDX, X-ray mapping, SEM, and VSM analysis. This nanomagnetic material was used as a catalyst to synthesize variously substituted polyhydroquinolines **52** without using a solvent and resulting in high yields (82–99 %). Additionally, it was also employed in cyclocondensation processes in ethanol, providing satisfactory to outstanding yields. The catalyst's heterogeneity was assessed by its exceptional reusability and hot-filtration test (Scheme 44).

**Scheme 45:**
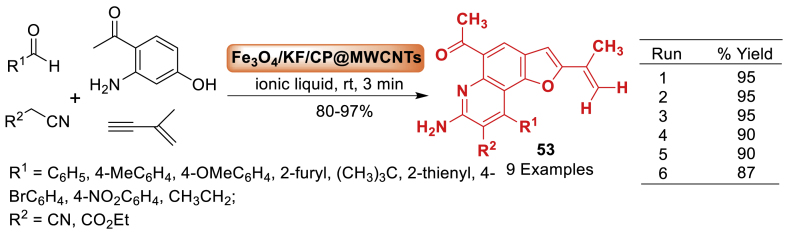
Synthesis of novel furo[2,3-*f*]quinoline.

Zareyee et al. [90] unfolded a multicomponent reaction of 2-amino-4-hydroxyacetophenone, isopropenylacetylene, aldehydes, and malononitrile or ethyl cyanoacetate in the presence of catalytic amounts of Fe_3_O_4_/KF/Clinoptilolite@MWCNTs magnetic nanocomposites using ionic liquid as green solvent at room temperature, new derivatives of furo[2,3-*f*]quinoline derivatives **53** were synthesized in high yields (80–97 %). This catalyst plays a major impact in the product yield and may be used multiple times in certain reactions. Because the synthesized molecules **53** have an NH group in structure, they exhibit strong antioxidant properties (Scheme 45).

**Scheme 46:**
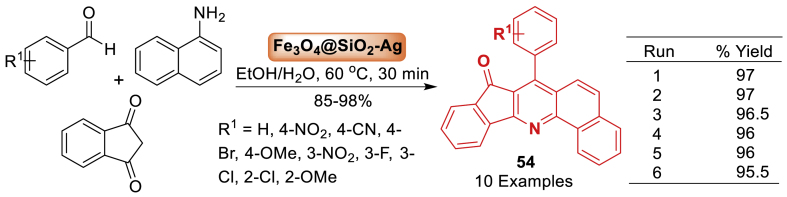
Scope of coupling reaction of substituted aromatic aldehyde, amine and 1,3-indanedione.

Next, in 2023, Singh et al. [91] investigated the design, synthesis, identification, and application of silver nanoparticles immobilized on Fe_3_O_4_. The performance of the new heterogeneous Fe_3_O_4_@SiO_2_-Ag nanocatalyst in the synthesis of quinoline derivatives **54** was assessed using a coupling reaction of aldehyde, amine, and 1,3-indanedione in water and ethanol at 60 ^o^C. The three-component reactions were carried out in heterogeneous circumstances, and the catalyst was successfully recycled six times with only a minor loss in activity. In addition to the benefits of a heterogeneous catalyst, this system makes silver nanoparticles an excellent candidate for organic reactions due to ease of use, mild reaction conditions, stability, and high efficiency (Scheme 46).

**Scheme 47:**
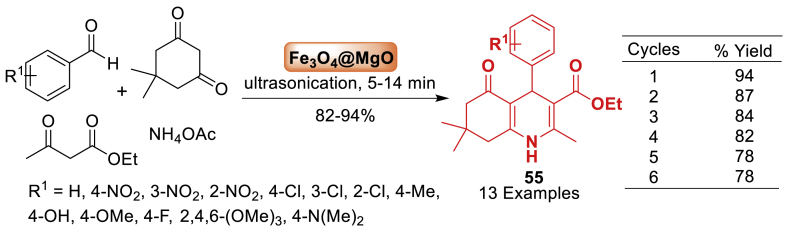
Synthesis of polyhydroquinoline derivatives in the presence of Fe_3_O_4_@MgO

The research group of Thakur [92] devised a two-fold co-precipitation approach to synthesize the core-shell nanostructured Fe_3_O_4_@MgO catalyst as illustrated in Scheme 47. High-resolution transmission electron microscopy (HR-TEM) scans revealed that the catalyst had a core-shell structure with a spherical shape. In the current study, Fe_3_O_4_@MgO is successfully exploited as an effective, unique, and recoverable nanocatalyst in an easy-to-follow, inexpensive, environmentally friendly, and productive approach for the synthesis of polyhydroquinoline derivatives **55** *via* a one-pot four-component Hantzsch condensation reaction. Importantly, magnetically retrievable Fe_3_O_4_@MgO nanoparticles exhibit great catalytic efficiency in solvent-free conditions and can be reused up to six times without substantial loss of catalytic activity. The report describes a greener way to achieve polyhydroquinoline **55** in a fast reaction time using ultrasonication.

#### 2.2.2 Role of organically dopped nanoparticles in the synthesis of quinoline derivatives

Here, this section provides an illustration of the investigation of organically dopped nanoparticles for the preparation of quinoline based molecular architecture and structures. Details that have been studied by various research groups have been provided in a manner that is easier to comprehend and understanding.

**Scheme 48:**
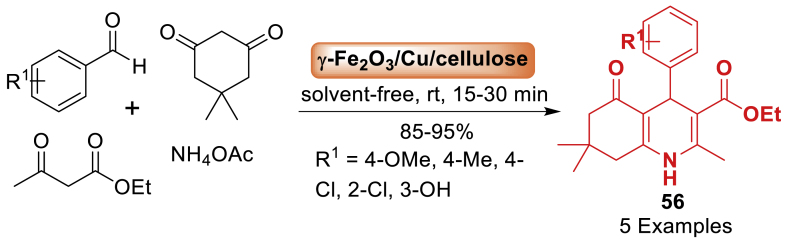
Synthesis of polyhydroquinoline derivatives in the presence of Fe_2_O_3_/Cu/cellulose as a nanocatalyst.

The Hantzsch 1,4-dihyropyridine synthesis is a multicomponent reaction that has been achieved using nano catalyst by Hamidi et al. [93] in 2014. This work described one-pot and four-component processes to synthesize 1,4-polyhydroquinoline derivatives **56** using a biopolymer-based *γ*-Fe_2_O_3_/Cu/cellulose nanocatalyst (Scheme 48). Importantly, the reaction accomplished under solvent free conditions at room temperature. The magnetic catalyst was highly efficient for the reaction and was conducted using a straightforward work-up process in eco-friendly manner in ethanol at ambient temperature.

**Scheme 49:**
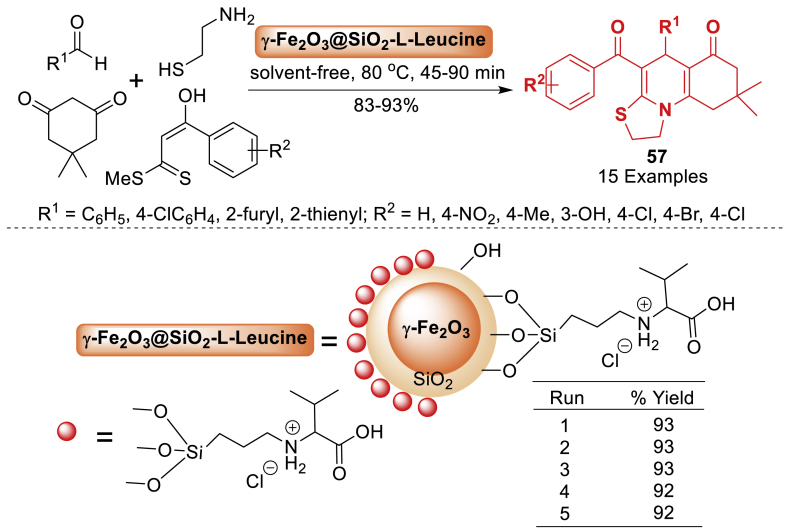
Substrate scope for the synthesis of thiazoloquinolines.

In 2016, the research group of Shaterian et al. [94] synthesized a novel superparamagnetic silica-encapsulated Fe_2_O_3_-supported L-Leucine. The synthesis of thiazoloquinolines **57** was accelerated by a four-component reaction involving *α*-enolicdithioesters, cysteamine, aromatic aldehydes, and dimedone under thermal solvent-free conditions, resulting in good to excellent yields (83–93 %) (Scheme 49). This unique superparamagnetic catalyst may be effortlessly isolated from the reaction mixture using an external magnet and reused at least five times without significant reduction in catalytic activity. Total 15 examples were prepared in this work by using electron donating and electron withdrawing decorated starting substrates.

**Scheme 50:**
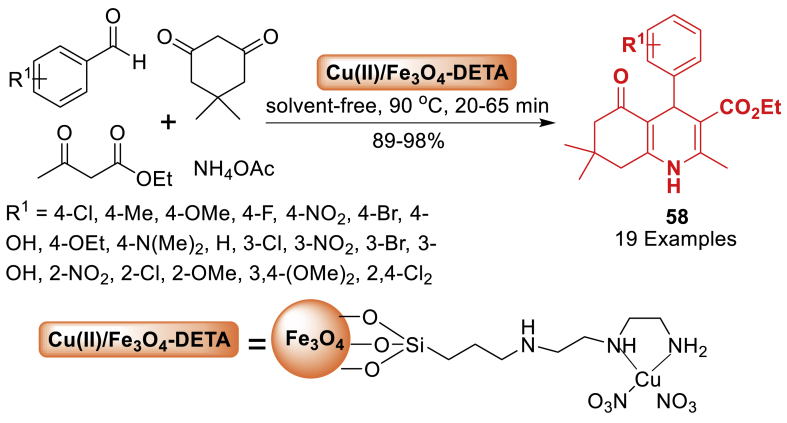
Synthesis of polyhydroquinolines in the presence of Fe_3_O_4_-DETA-Cu(II).

In 2017, Kazemi et al. [95] synthesized a novel magnetically separable catalyst composed of a Cu (II) complex immobilized on Fe_3_O_4_ nanoparticles functionalized with diethylenetriamine (Fe_3_O_4_-DETA Cu(II)). The produced nanosolid catalyst proved to be an effective heterogeneous catalyst for achieving polyhydroquinoline derivatives **58** without the use of any solvent. The reaction of benzaldehyde, dimedone, *β*-diketoester and ammonium acetate delivered final product **58** in 89–98 % yields within 20–65 min. The catalyst was easily recovered from the final product by magnetic decantation and could be reused for at least seven reactions with minimal loss of catalytic activity (Scheme 50).

**Scheme 51:**
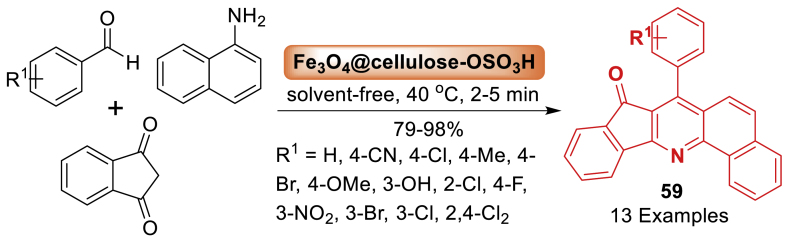
Preparation of 7‐aryl‐8*H*‐benzo[*h*] indeno[1,2‐*b*]quinoline‐8‐one derivatives.

An eco-friendly and cost-effective sulfonated magnetic cellulose-based nanocomposite was utilized as a catalyst for the rapid synthesis of 7-aryl-8*H*-benzo[*h*]indeno[1,2-*b*]quinoline-8-ones **59** from 1,3-indanedione, aromatic aldehydes, and 1-naphthylamine by Yeganeh and co-workers [96]. The reaction occurred under solvent-free conditions, resulting in high yields (79–98 %) in short reaction times (2–5 min) as depicted in Scheme 51. The nano-bio-structure catalyst can be effortlessly isolated from the reaction mixture with an external magnet and can be reused multiple times. Total 13 examples were prepared in this work by the authors.

**Scheme 52:**
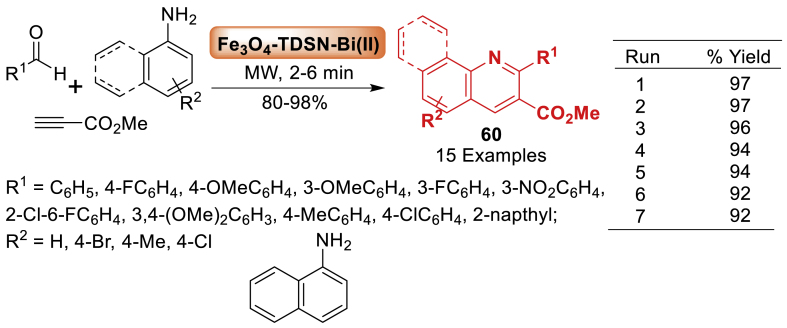
Synthesis of quinoline fused derivatives in the presence of Fe_3_O_4_-TDSN Bi(III).

In this report, Rudbari et al. [97] employed Fe_3_O_4_-TDSN-Bi(III) as a very effective and recyclable catalyst for the selective synthesis of quinoline derivatives **60** from arylamines, arylaldehydes, and methyl propiolate in a single step using microwave radiation and without the need for a solvent. The one-pot reaction involved benzaldehyde, aniline and terminal alkyne to afford the quinoline derivatives **60** in 80–98 % yields as illustrated in Scheme 52. This process is characterized by atom-economy, good to excellent yields, high diversity, facile work up, and simple catalyst recovery and reusability.

**Scheme 53:**
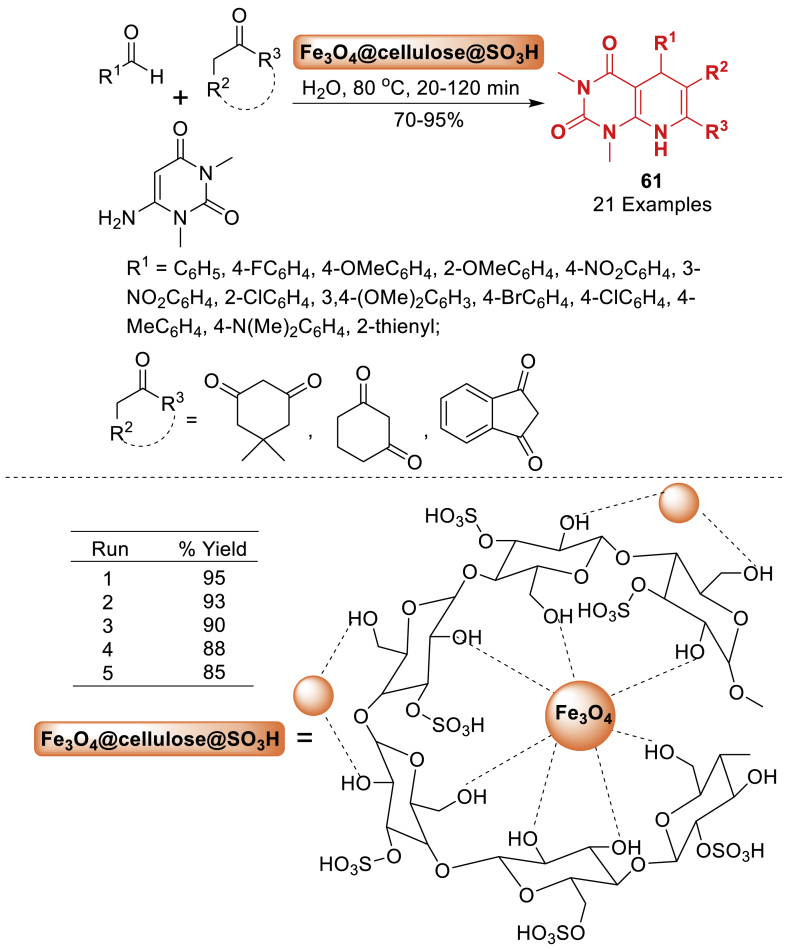
Synthesis of diversified pyrimido[4,5-*b*]quinolines.

Under this report, Sabaqian et al. [98] synthesized cellulose-based magnetic nano-composite with supported SO_3_H group. Thereafter, its potential to facilitate the one-pot, three-component synthesis of pyrimido[4,5-*b*]quinolone derivatives **61** was then assessed as mentioned in Scheme 53. The reaction was accomplished by using benzaldehyde, carbonyl compounds and 2-aminopyrimidine in the presence of green solvent (H_2_O) at 80 ^o^C. The biopolymer-based catalyst can be easily recovered and reused multiple times without any reduction in yield.

**Scheme 54:**
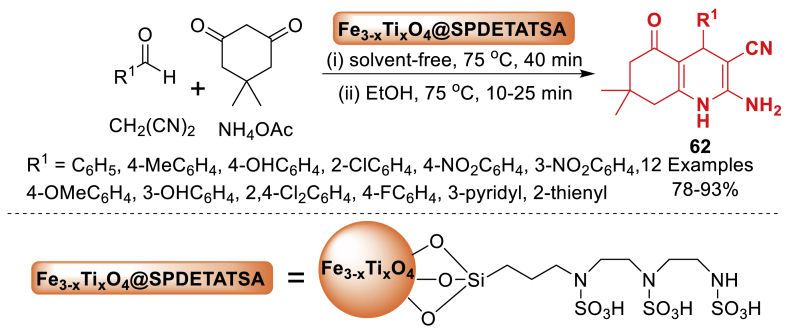
One-pot operation for the synthesis of hexahydroquinolines.

In 2017, Badalkhani et al. [99] introduced the grafting of *N*‐(3‐silyl propyl) diethylene triamine *N*, *N'*, *N″*-tri‐sulfonic acid onto magnetic Fe_3-x_Ti_x_O_4_ nanoparticles for the first time (Scheme 54). The results verified the effective immobilization of sulfamic acid groups onto the magnetic support. Further, the nanoparticles showed strong catalytic activity as a new magnetically reusable acid nanocatalyst in achieving various hexahydroquinolines **62** through one-step tandem reactions with great efficiency. Total 12 examples were achieved in 78–93 % yields. The nanocatalyst maintained its catalytic activity well for the formation of reaction products after four rounds of recycling without any significant loss of activity.

**Scheme 55:**
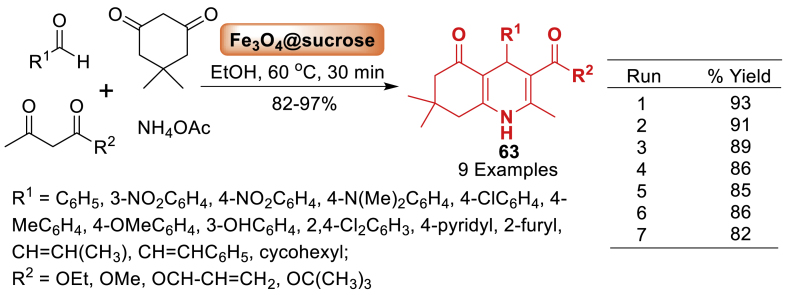
Method for the synthesis of polyhydroquinoline *via* Fe_3_O_4_-sucrose as a nanocatalyst.

Next, in 2018, Heydari and co-workers [100] conducted a straightforward synthesis of penta-substituted polyhydroquinolines (PHQs) **63** using environment-friendly conditions and a new nano catalyst. Products **63** have been synthesized from two distinct types of 1,3-dicarbonyls, conjugated aldehydes, and ammonium acetate. Magnetic nanoparticles (MNPs) with a core-shell structure were synthesized utilizing table sugar as a cost-effective carbohydrate. In addition to its commercial and semi-industrial applications, the reusability of magnetic catalysts has generated significant interest as a substitute for traditional homogeneous promoters. Pleasingly, over 78 % of the initial performance has resulted in a significant overall yield after the seventh cycle (Scheme 55).

**Scheme 56:**
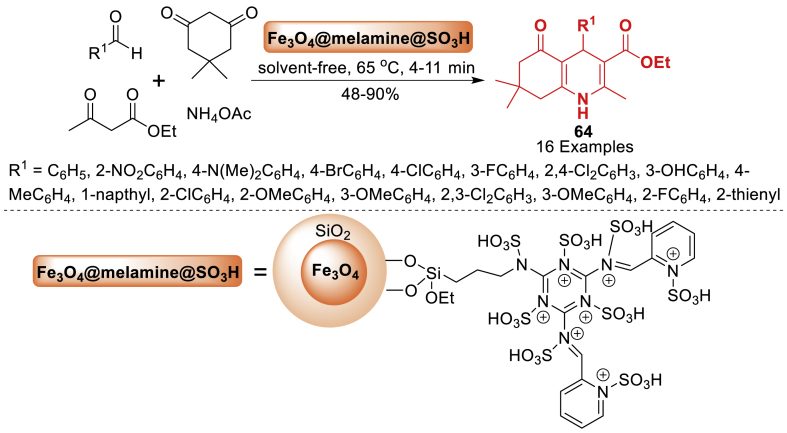
Solvent-free reaction for the synthesis of quinoline tethered derivatives.

Under this work, Seyf et al. [101] reported a novel Brønsted acid nano-magnetic supported ionic liquid catalyst. This catalyst worked as a solid heterogeneous catalyst. The catalyst activity was evaluated in the synthesis of hexahydroquinoline derivatives **64** by a four-component reaction including dimedone, aldehyde, ethyl acetoacetate, and ammonium acetate, resulting in shortened process time (Scheme 56). Total 16 examples were achieved in 48–90 % yields. The suggested technique is characterized by being solvent-free, metal-free, high-yielding, with a reduced reaction time (4–11 min), fast recovery of catalyst, and a simple workup procedure. Moreover, the electron donating and electron withdrawing group decorated benzaldehyde gave good results for the present reaction.

**Scheme 57:**
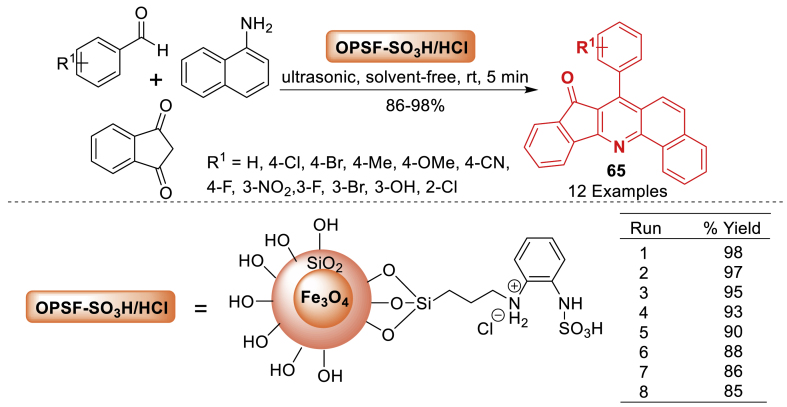
Three-component reaction for the preparation of 7-aryl-8*H*-benzo[*h*]indeno[1,2-*b*]quinoline-8-one derivatives.

**Scheme 58:**
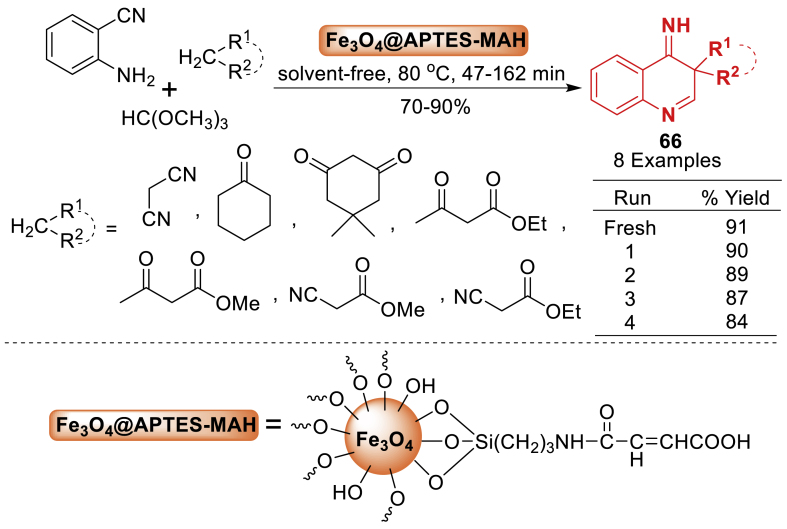
Fe_3_O_4_@APTES-MAH-mediated synthesis of 4-iminoquinolines.

Firouzi-Haji’s group [102] generated a new hybrid magnetic organometallic nanocatalyst by stabilizing *o*-phenylenediamine on silica-coated Fe_3_O_4_ magnetic nanoparticles (Scheme 57). The scanning electron microscope (SEM) picture of the prepared nanocatalyst revealed a core-shell spherical shape with a consistent size distribution, averaging around 40 nm. The catalytic activity was examined for the selective synthesis of 7-aryl-8*H*-benzo[*h*]indeno[1,2-*b*]quinoline-8-one derivatives **65** with good to excellent yields (86–98 %) as displayed in Scheme 58. Notably, this was achieved under solvent-free conditions and ultrasonic irradiation at room temperature.

Nasr-Esfahani’s team [103] synthesized a novel 4-iminoquinolines **66** by reacting 2-aminobenzonitrile, orthoesters, and active methylenes with Brønsted acid nanocatalysts (Fe_3_O_4_@APTES-MAH NPs or VSA NRs) in solvent-free conditions. The nano catalyst system was synthesized by reacting chlorosulfonic acid with sodium monovanadate to achieve high purity. Fe_3_O_4_@APTES NPs were fabricated by coating Fe_3_O_4_ nanoparticles with 3-aminopropyltriethoxysilane. The reaction is distinguished by its high yields, high diversity, easy product purification, low cost, short reaction durations, catalyst reusability, and stability (Scheme 58).

**Scheme 59:**
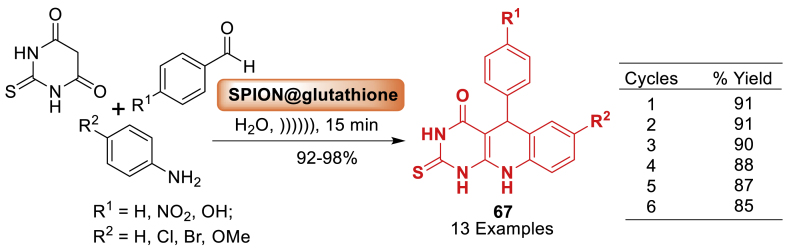
Ultrasonic-promoted preparation of arylpyrimido-[4,5-*b*]quinoline-diones using SPION@glutathione

Glutathione's potential as a catalyst for carbon-oxygen ring cyclization was studied by the team of Nongkhlaw [104] through the experimental and computational methods as described in Scheme 59. Glutathione molecules were fixed onto superparamagnetic iron-oxide nanoparticles to enhance the catalyst's effectiveness and facilitate magnetic-assisted reusability. The performance of new catalyst was investigated on the synthesis of quinoline frameworks **67**. It is found that catalyst delivered final product **67** in good to excellent yields (92–98 %) within 15 min only. The catalytic ability in achieving various indolyl chromenes, pyrrolyl chromenes, and indoloxanthenes was thoroughly studied. The synthetic protocol was determined to be efficient, cost-effective, and nature friendly.

**Scheme 60:**
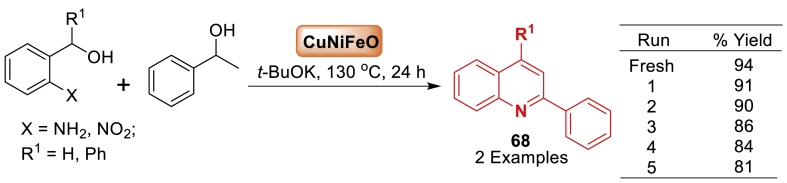
Synthesis of quinoline substituted derivatives.

Next, in 2019, Bhanage et al. [105] described the formation of nano CuNiFeO and its use in the synthesis of quinolines **68** (Scheme 60). The heterogeneous catalyst Nano CuNiFeO demonstrates remarkable efficiency in catalysing the double dehydrogenation tandem cyclization reaction of 2-amionbenzyl alcohol with alcohols, leading to a straightforward, sustainable, and environment-friendly synthesis of quinolines **68**. The nanocatalyst's magnetic characteristic allowed for easy separation from the reaction mixture using an external magnet and enabled reusability for up to five cycles. This is the first report of a heterogenous magnetic CuNiFeO nanocatalyst being used for this reaction.

**Scheme 61:**
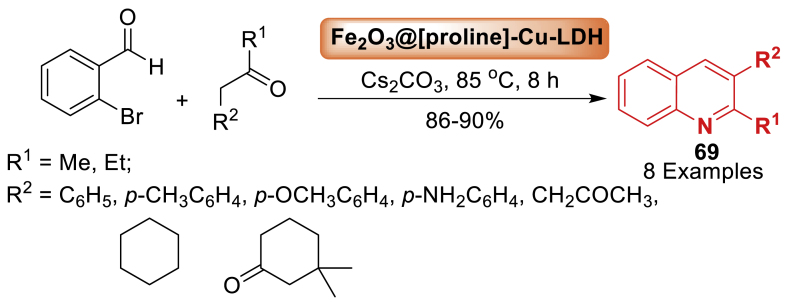
One-pot synthesis of quinolines.

An effective bifunctional catalytic system, Fe_2_O_3_@[proline]-CuMgAl-L(ayered)D(ouble) H(ydroxide), was fabricated with a new magnetic core-shell structure by Heydari’s team [106] in 2020. L-proline was intercalated between LDH layers and Cu (II) was coupled with Mg and Al in the LDH structure. This allowed for the simple synthesis of significant pharmaceutical *N*-aryl-substituted heterocyclic compounds; quinolines **69**. To avoid poisonous solvents and dangerous azidation reagents, a simple procedure was used in this regard by the author (Scheme 61). The use of green media, lowering the amount of starting reagents needed, high yields (86–90 %), and providing a short reaction time and lower temperature were all improved by these procedures.

**Scheme 62:**
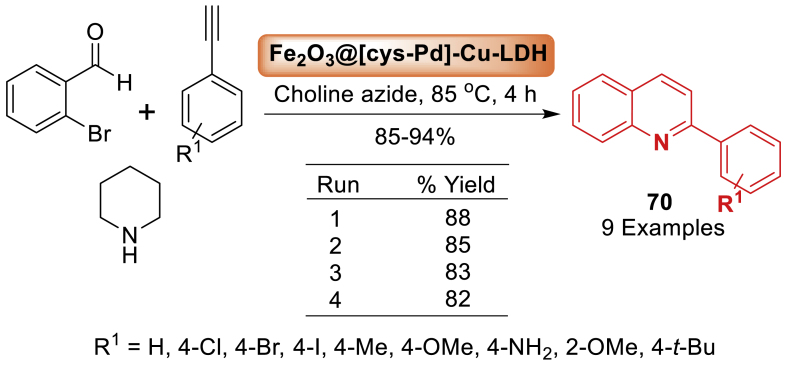
2-Substituted quinoline synthesis in the presence of *γ*-Fe_2_O_3_@Cu-LDH@Cysteine-Pd

Next, the magnetic *γ*-Fe_2_O_3_@Cu-LDH@Cysteine-Pd dual nanocatalyst system also combined MNPs and LDH to enhance separation and catalytic properties by Heydari and team [107] (Scheme 62). In this context, a simple surface modulation by replacing copper (II) metal cations with LDH cations was achieved. Furthermore, the interface defect structure of LDH was altered by the immobilization of a Pd (II) complex as an active metal catalyst. To demonstrate catalytic efficiency, the synthesized biocompatible catalyst was used in an affordable method of quinoline **70** synthesis that included A^3^-coupling, C-N coupling, and intramolecular cyclization. Quinoline derivatives **70** were afforded using a low-cost and readily available starting material; 2-bromo benzaldehyde, and *γ*-Fe_2_O_3_@Cu-LDH@Cysteine-Pd. It is noted that total 9 examples were prepared in 85–94 % yields.

**Scheme 63:**
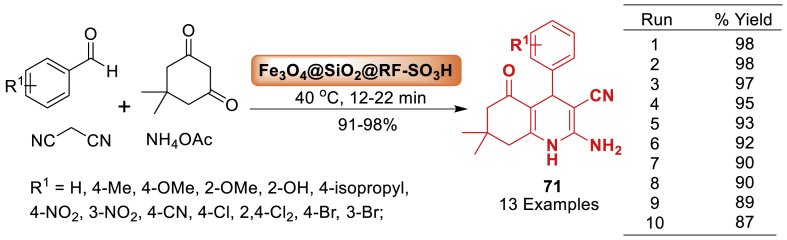
Fe_3_O_4_@SiO_2_@RF-SO_3_H-catalysed synthesis of hexahydroquinoline derivatives under solvent-free condition.

Here, a novel spherically shaped core@double-shell acidic nanocatalyst (Fe_3_O_4_@SiO_2_@RF-SO_3_H) [RF: resorcinol-formaldehyde resin] was prepared by Beni et al. [108]. The concentration of H^+^ on the Fe_3_O_4_@SiO_2_@RF was found to be 1.3 mmol g^−1^. The well-defined Fe_3_O_4_@SiO_2_@RF-SO_3_H core-shell heterostructures demonstrated high stability, efficient recyclability (10 cycles), and catalytic activity in a one-pot condensation reaction of aromatic aldehydes, dimedone, malononitrile, and ammonium acetate to afford hexahydroquinoline derivatives **71**. Notably, total 13 examples were prepared in excellent yields (91–98 %) within 12–22 min as discussed in Scheme 63.

**Scheme 64:**
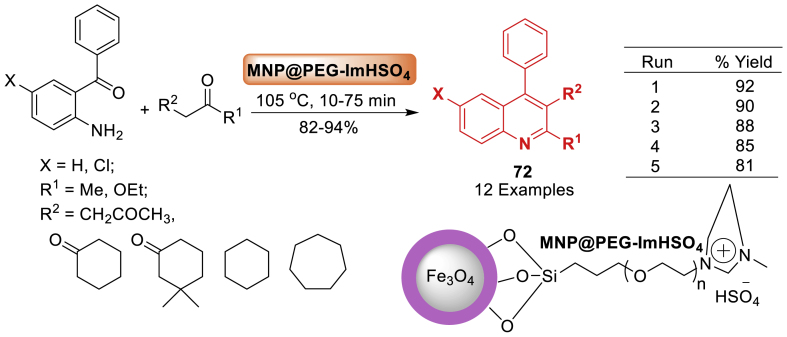
MNP@PEG-ImHSO_4_-mediated synthesis of substituted quinolines.

In 2022, a new acidic imidazolium-based ionic liquid supported on magnetic nanoparticles (MNP@PEG-ImHSO_4_) was synthesized by Banitaba’s research group [109]. Its catalytic activity was then examined in the Friedlander synthesis of quinolines **72**, which involved a condensation reaction between 2-aminoaryl ketones and *α*-methylene ketones under solvent-free conditions. Total 12 examples were prepared in 82–94 % yields within short span of reaction time by the author. The catalyst could be easily removed from the reaction mixture using an external magnet and reused in subsequent reactions with no noticeable loss of activity (Scheme 64).

**Scheme 65:**
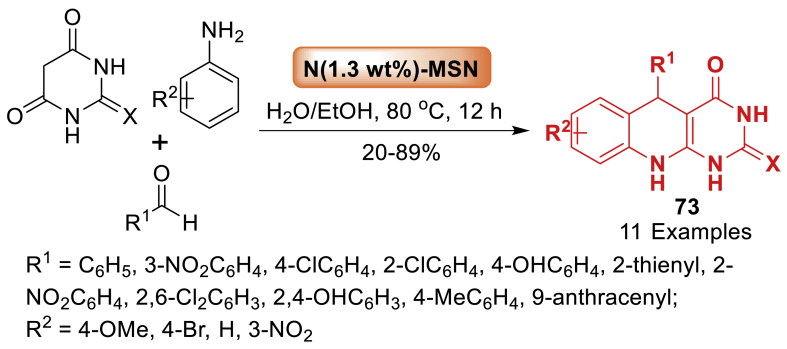
Synthesis of arylpyrimido[4,5-*b*]quinolone-dione derivatives.

A new acidic imidazolium-based ionic liquid supported on magnetic nanoparticles (MNP@PEG-ImHSO_4_) was fabricated and examined for the first time by Sun et al. [110]. Later, using a solvent-free condensation reaction between 2-aminoaryl ketones and *α*-methylene ketones, its catalytic activity was examined in the Friedlander synthesis of quinolines **73**. Using an external magnet, the catalyst was easily removed from the reaction mixture and could be reused in consecutive reactions without experiencing a significant reduction in activity. Total 21 examples were prepared in 20–89 % yields (Scheme 65).

**Scheme 66:**
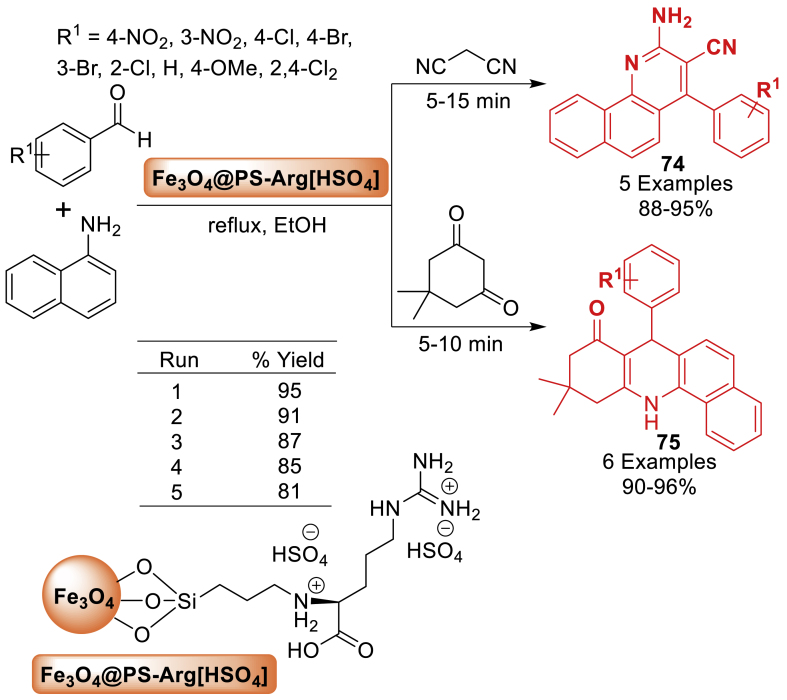
Reaction of substituted benzaldehyde with aromatic anilines.

In this research, Shariati’s group [111] disclosed a method to fabricate functionalize Fe_3_O_4_ magnetic nanoparticles, and arginine. Fe_3_O_4_@PS-Arginine magnetic nanoparticles that were manufactured were altered to synthesize Fe_3_O_4_@PS-Arg[HSO_4_]. For the production of 2-amino-4-arylbenzo[*h*] **74**, **75**, these nanoparticles were employed as an environmentally acceptable solid acid magnetic nanocatalyst.10.10-dimethyl-7-aryl-9,10,11,12-tetrahydrobenzo[*c*] **75** and quinoline-3 carbonitrileacridin-8(7*H*)-one derivatives **74** by means of a one-pot synthesis involving aromatic aldehydes and *α*-naphthilamine with either dimedone or malononitrile (Scheme 66). The main benefits of utilizing this catalyst are its easy operation, high reaction yields (88–96 %), multiple reusability, fast reaction time (5–15 min), and simple separation from reaction mixture.

**Scheme 67:**
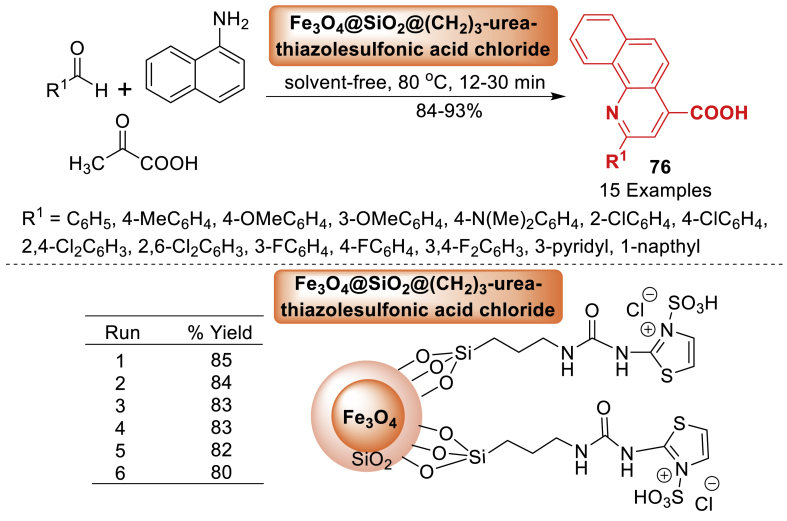
Catalytic synthesis of 2-aryl-quinoline-4-carboxylic acids.

In this investigation, Torabi et al. [112] presented the design and synthesis of Fe_3_O_4_@ SiO_2_@(CH_2_)_3_-urea-thiazole sulfonic acid chloride, a novel class of ionically tagged magnetic nanoparticles with urea linkers. Aryl aldehyde, pyruvic acid, and 1-naphthylamine assembled starting materials in the presence of the innovative reusable catalyst to afford the 2-aryl-quinoline-4-carboxylic acid derivatives **76** as depicted in Scheme 67. Total 15 examples were prepared in 84–93 % yields. The current methodology has several advantages such as short reaction time (12–30 min), solvent-free, reusable catalyst, high yield and high diversity.

**Scheme 68:**
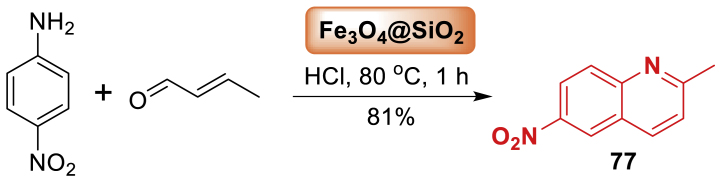
Synthesis of nitro-substituted quinoline.

Thereafter, in 2021, the report of Pullela et al. [113] described the usage of Fe_3_O_4_@SiO_2_ in the synthesis of quinoline derivatives **77**. The reaction of 4-nitroaniliune and *α*,*β*-unsaturated carbonyl compounds delivered the targeted framework **77** in 81 % yield as shown in Scheme 68. The reduction in reaction time due to surface-enabled catalysis of nanoparticles is 110 to 80 min. Interestingly, the inclusion of a catalyst quadrupled the reaction yield. This near-homogeneous catalysis of 40 nm-sized, silica-functionalized magnetite nanoparticles has far-reaching applications in the pharmaceutical industry for medications such as chloroquine and hydroxychloroquine, the two important treatments for prophylactic COVID-1 treatment.

**Scheme 69:**
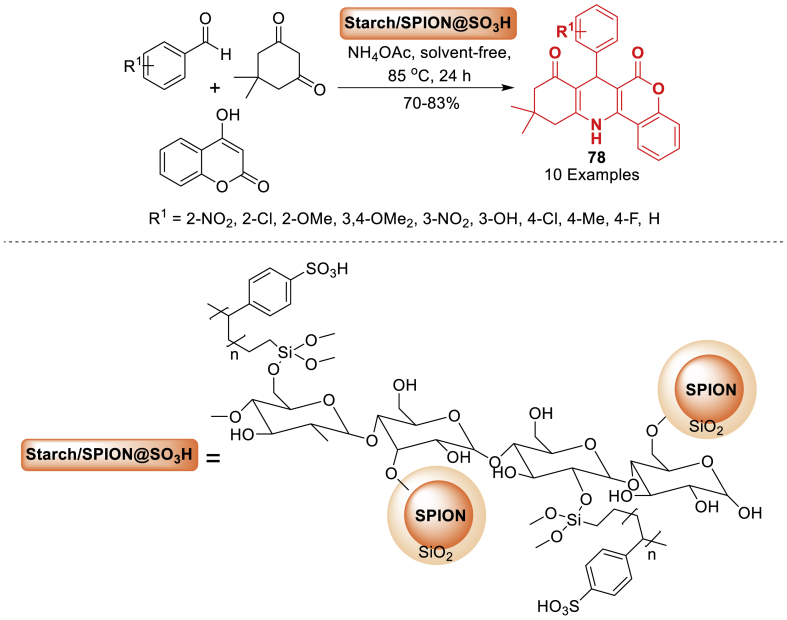
Synthesis of chromeno[4,3-*b*]quinoline-6,8(9*H*)-dione in the presence of Starch/SPION@SO_3_H

The group of Bahadorikhalili et al. [114] disclosed a, sulfonic acid functionalized magnetic starch (Starch/SPION@SO_3_H) based nano catalyst has been synthesized. Magnetic starch (Starch/SPION) is fabricated by covalently modifying starch with silica-capsulated superparamagnetic iron oxide nanoparticles (SPION). In the multicomponent reaction of 4-hydroxycoumarin, benzaldehydes, dimedone, and ammonium acetate for the synthesis of chromeno[4,3-*b*]quinoline-6,8(9*H*)-dione **78**, the catalytic activity of Starch/SPION@SO_3_H is assessed. In the synthesis of derivatives of chromeno[4,3-*b*]quinoline-6,8(9*H*)-dione **78**, the catalyst exhibits extremely good activity (Scheme 69). A variety of benzaldehydes with electron donating and withdrawing groups are employed as the initial substrates, and each of them gives the intended products in high isolated yields.

**Scheme 70:**
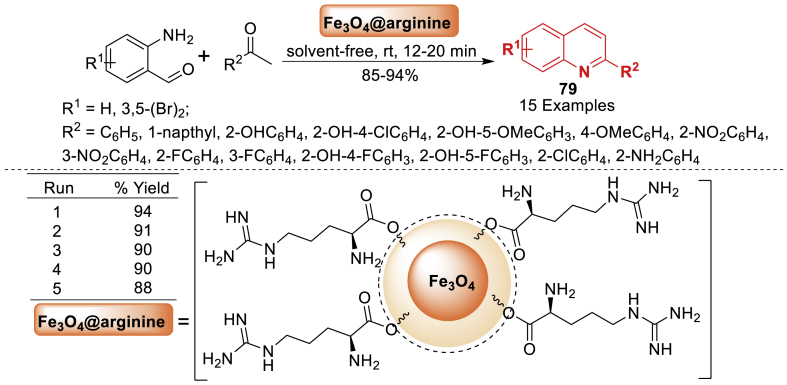
Synthesis of quinoline derivatives using Fe_3_O_4_@arginine

Under this investigation, Siddiqui et al. [115] reported a highly efficient organocatalyst arginine-coupled magnetic nanoparticles as described in Scheme 70. The results show that the catalyst is suitable for a broad variety of functional groups and generates several quinoline derivatives **79** in high yields (85–94 %) without the use of a solvent. The catalyst's superior activity can be enhanced by the synergistic effect of the following factors: Iron oxide nanoparticles as supports offer significant surface areas, high surface-to-volume ratio, enhanced reactivity and selectivity, and reduced energy consumption. Additionally, utilizing the environment-friendly, non-toxic, and cost-effective L-arginine amino acid as a coating agent requires easy handling and storage.

**Scheme 71:**
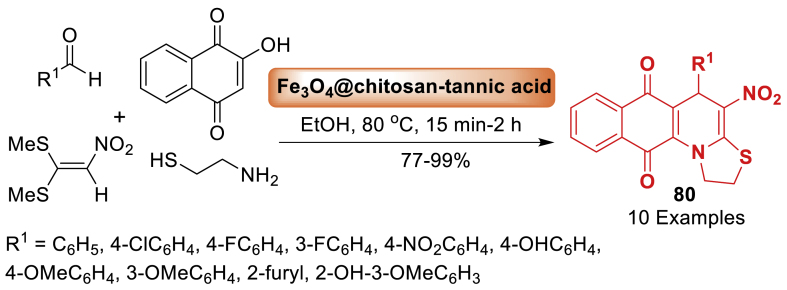
Catalytic activity of Fe_3_O_4_@CS-TA bionanocomposite for the synthesis of benzo[*g*]thiazolo[3,2-*a*]quinolones.

Further, in 2023, Maleki’s group [116] unfolded a route to fabricate an efficient magnetic bio-nano-composite and protect ferrite nanoparticles from oxidation and aggregation, the prepared Fe_3_O_4_ was supported by chitosan and tannic acid as the first and second coating layers, respectively. Moreover, the catalytic activity was tested by synthesizing 4-nitro-5-phenyl-1,2-dihydro-5*H*-benzo[*g*]thiazolo[3,2-*a*]quinolines-6,11-dione **80** with potent antitumor activity from *β*-nitro thiazolidine, 2-hydroxy-1,4-naphthoquinone, and various aromatic aldehydes using an aza-ene reaction followed by intramolecular cyclization. Some of the most notable advantages of this technique include the ability to afford products **80** in short reaction times (5–14 min) with high yields (82–94 %), the catalyst's environmentally friendly nature, and the ease with which the catalyst can be separated and recycled due to the presence of the superparamagnetic core (Scheme 71).

#### 2.2.3 Role of hybrid nanoparticles in the synthesis of quinoline derivatives

This section is an illustration of the reports conducted for the preparation of quinoline containing molecular framework by using hybrid nanoparticles that possess magnetic properties. The information that has been examined by numerous research groups globally has been presented in a manner that is more accessible and comprehensible.

**Scheme 72:**
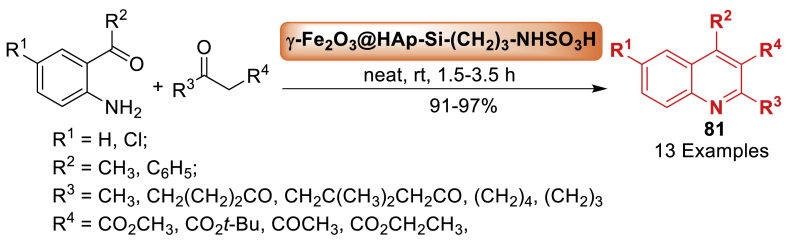
Friedlander reaction of 2-amino aryl ketones with ketones in the presence of nanocatalyst.

In 2011, Heydari et al. [117] implemented an innovative and environment-friendly approach for the organic synthesis as shown in Scheme 72. The efficiency of catalyst was checked in the synthesis of quinoline derivatives **81** by the reaction of substituted aniline and carbonyl compounds at room temperature. The reaction delivered the final products **81** in 91–97 % yields. The catalyst Fe_2_O_3_-HAp-(CH_2_)_3_-NHSO_3_H consists of magnetic nanoparticles supporting propylsulfamic acid placed onto hydroxyapatite. It is a very efficient heterogeneous acid catalyst that may be reused for at least ten reaction cycles without a notable decrease in their activity.

**Scheme 73:**
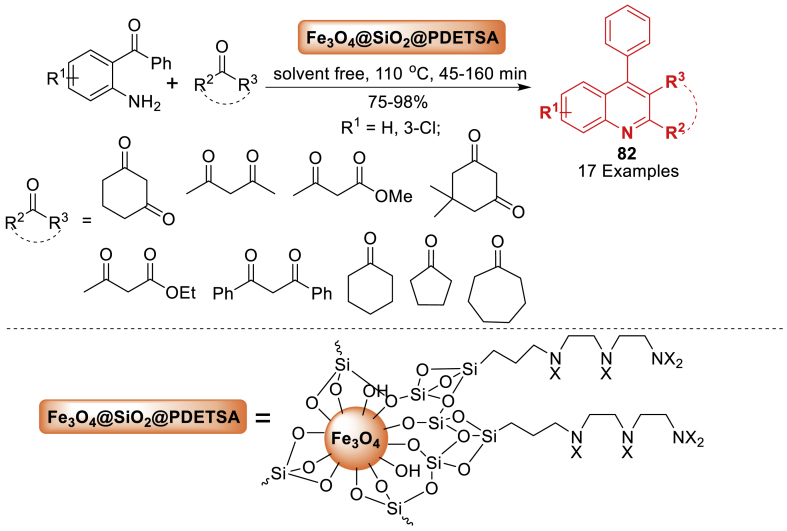
Synthesis of quinolines under solvent-free reaction conditions.

In 2014, Samieadel’s team [118] reported the synthesis of sulfamic acid supported on Fe_3_O_4_@SiO_2_ superparamagnetic nanoparticles as a solid acid catalyst with a high density of sulfamic acid groups. The structural and magnetic characteristics of functionalized Fe_3_O_4_@SiO_2_ nanoparticles are determined using TEM, IR, VSM, XRD, TGA, and elemental analysis. It is interesting to note that the synthesized nanoparticles were investigated as a recyclable acidic catalyst for synthesizing quinoline derivatives **82**, an important class of potentially therapeutic candidates (Scheme 73). The products **82** are achieved in good to excellent yields (72–98 %) by a one-pot reaction process that combines carbonyl compounds with 2-amino benzophenone without the use of solvents.

**Scheme 74:**
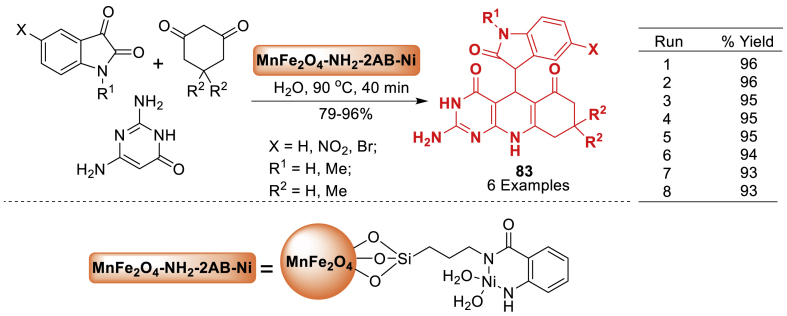
Nanoparticle-catalysed reaction for the synthesis of 20-amino-80,80-dimethyl 80,90-dihydro-30*H*-spiro[indoline-3,50-pyrimido[4,5-*b*]quinoline]-2,40,60 (70*H*,100*H*)-triones

In this article, the research group of Ghahremanzadeh [119] developed superparamagnetic manganese ferrite nanoparticles using a co-precipitation approach and then coated with 3-aminopropyltriethoxysilane (APTES) by a silanization reaction. The nanoparticles' catalytic properties were assessed by synthesizing spirooxindoles-quinoline molecular hybrids **83** in water using a green method, demonstrating outstanding catalytic performance. The final products **83** were obtained in 79–96 % yields as mentioned in Scheme 74. The catalyst was easily recovered by using an external permanent magnet. The catalyst was reused for fresh reactions without a notable decrease in catalytic activity.

**Scheme 75:**
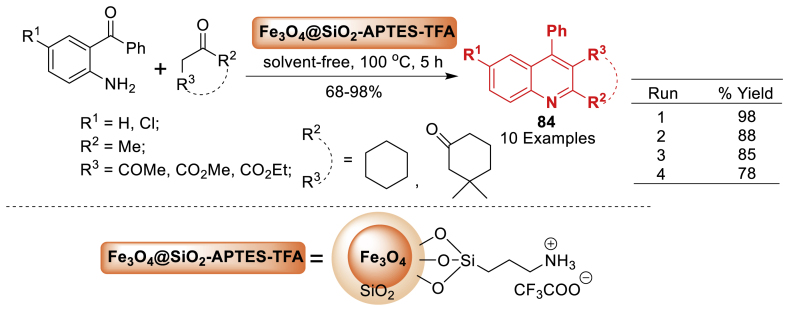
Fe_3_O_4_@SiO_2_/APTES-TFA-mediated synthesis of quinolines.

As discussed in Scheme 75, a magnetically recoverable nanocatalyst with trifluoroacetic acid immobilized on Fe_3_O_4_@SiO_2_ APTES core-shell structure was synthesized by Sepahvand’s group [120] in 2015. This catalyst contains a strong Brönsted acid, was effective in catalysing the Friedländer synthesis of quinolines **84** using different cyclic and acyclic dicarbonyl compounds in a solvent-free environment. In addition to this, the current protocol delivered the final products **84** in 68–98 % yields. The current protocol also compatible with electron donating and electron withdrawing group decorated starting precursors.

**Scheme 76:**
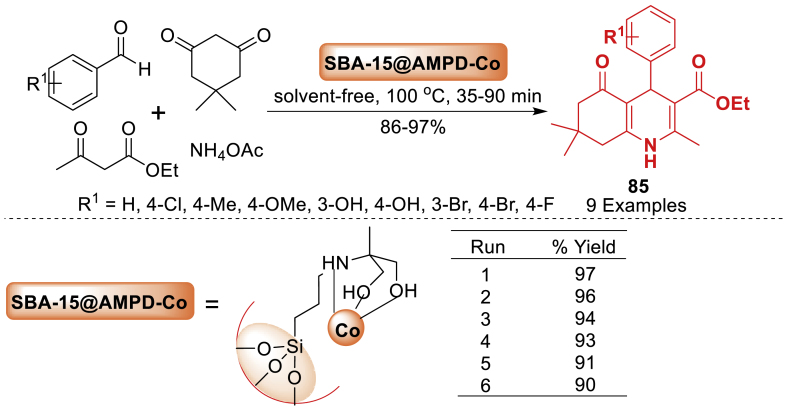
Hantzsch reaction for the synthesis of different polyhydroquinolines.

In 2018, a green and novel catalyst was fabricated by Ghadermazi and co-workers [121] immobilizing a cobalt complex on the mesostructured SBA-15 surface. The mesostructural material served as a highly effective and environment-friendly interphase catalyst for oxidizing processes and generating polyhydroquinoline derivatives **85** (Scheme 76). All the reactions were conducted rapidly and resulted in high yields (86–97 %). Additionally, the catalyst can be utilized for up to six cycles without experiencing notable deterioration in its catalytic efficiency or cobalt loss.

**Scheme 77:**
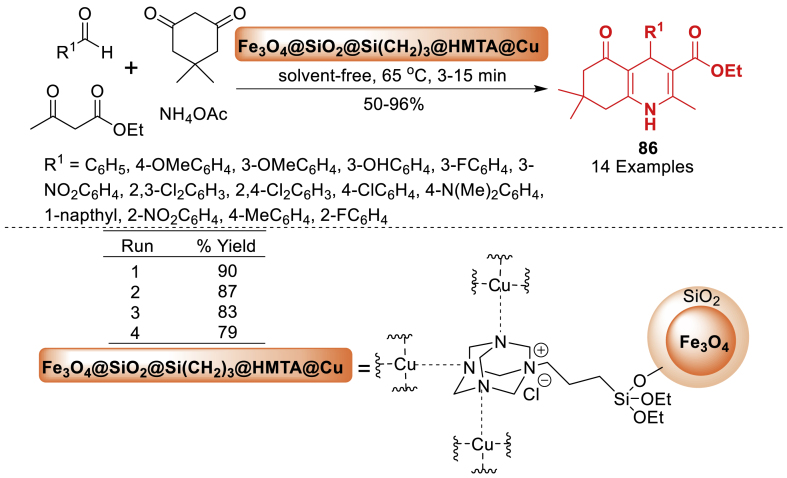
Synthesized different HHQ derivatives under the optimum condition.

In 2019, a nanomagnetic catalyst utilizing a nano-Fe_3_O_4_ core was fabricated and employed in the synthesis of hexahydroquinoline derivatives **86** as a solid heterogeneous catalyst by Gilan et al. [122]. Catalyst efficiency was evaluated in the synthesis of hexahydroquinoline (HHQ) derivatives **86** (Scheme 77). The present technique is characterized by solvent-free, high-yields (50–96 %), with a reduced reaction time (3–15 min), facile synthesis and separation of the catalyst, and an efficient workup procedure. The optimization process determined that the best reaction temperature and catalyst amount were 65 °C and 0.05 g, respectively.

**Scheme 78:**
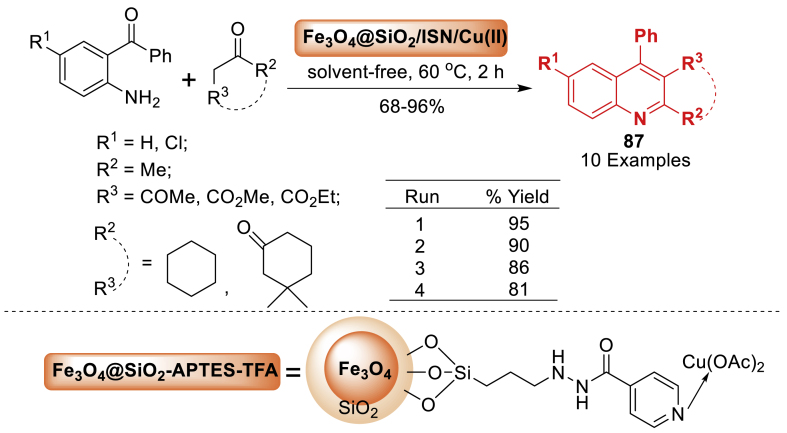
Friedländer synthesis of quinoline derivatives through Fe_3_O_4_@SiO_2_/ISN/Cu(II)

In another study in 2019, Rahimi and co-workers [123] described a method to efficiently synthesize Fe_3_O_4_@SiO_2_/Isoniazid/Cu(II) as a reusable magnetic nanocatalyst as shown in Scheme 78. Further, the nanocatalyst was utilized in the Friedländer synthesis to efficiently synthesize quinoline derivatives **87** from *α*-methylene ketones and 2-aminoaryl ketones under mild reaction condition. This process offers short reaction times, high yields (68–96 %), and easy catalyst separation using an external magnet and solution decanting. Importantly, the catalyst can be recovered for several cycles, maintaining its catalytic activity for up to four uses.

**Scheme 79:**
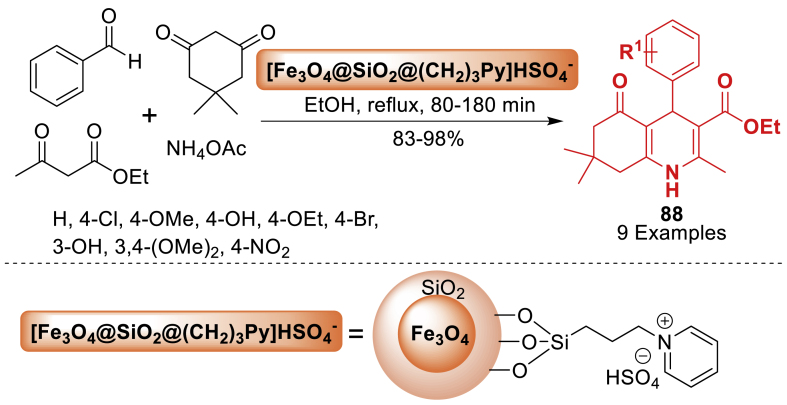
Synthesis of polyhydroquinolines using [Fe_3_O_4_@SiO_2_@(CH_2_)_3_Py]HSO_4_

The synthesis of polyhydroquinoline derivatives **88** was achieved by the team of Azmoudehfard [124] through a four-component condensation reaction involving aromatic aldehydes, dimedone, ethyl acetoacetate, and ammonium acetate as displayed in Scheme 79. This reaction took place in the presence of a catalytic quantity of ionic liquid on silica-coated Fe_3_O_4_ nanoparticles, serving as a heterogeneous, recyclable, and highly effective catalyst. The desired products **88** were obtained in good to excellent yields (83–98 %) in ethanol under reflux conditions.

**Scheme 80:**
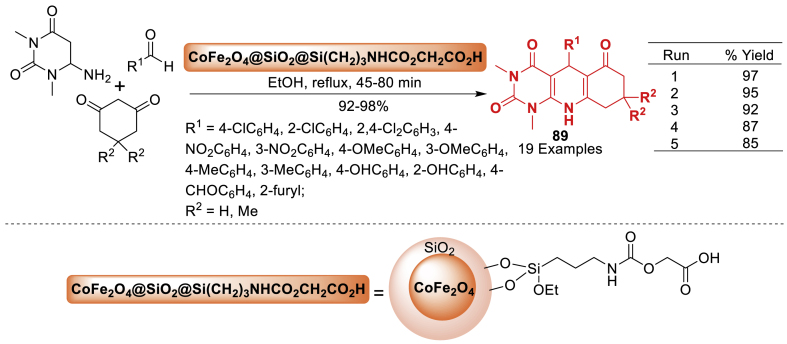
Synthesis of pyrimido[4,5-*b*]quinoline derivatives.

In 2020, Nikpassand et al. [125] developed a one-pot synthesis of pyrimido[4,5-*b*]quinoline derivatives **89** through a three-component reaction involving aldehydes, 6-amino-1,3-dimethyluracil, and 1,3-dicarbonyl compounds. The current methodology let to final products **89** in 92–98 % yields. This reaction took place in the presence of a novel magnetic catalyst, glycolic acid-supported cobalt ferrite CoFe_2_O_4_@SiO_2_@Si(CH_2_)_3_NHCOOCH_2_COOH, in ethanol under reflux conditions. The products **89** were obtained efficiently in high yields within suitable reaction durations (45–80 min) under eco-friendly circumstances (Scheme 80). This approach offers notable advantages such as high efficiency and simple catalyst isolation using an external permanent magnet.

**Scheme 81:**
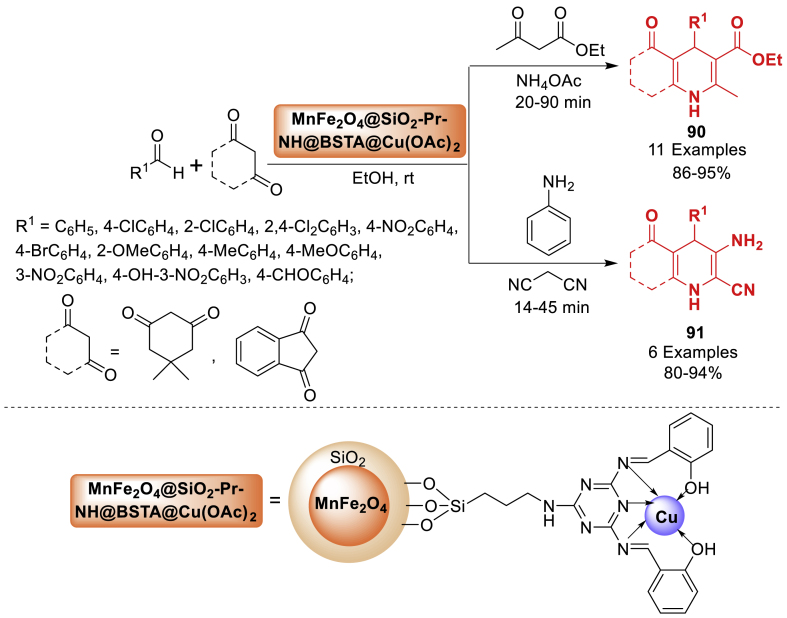
MNP@BSAT@Cu(OAc)_2_-assisted synthesis of 1,4-dihydropyridines and *N*-aryl quinolines.

Under this research, the group of Bodaghifard [126] presented the design and synthesis of a heterogeneous catalyst by functionalizing manganese ferrite nanoparticles enclosed in a silica film with Schiff base and then adding copper (Scheme 81). The organic-inorganic hybrid material was effectively utilized as a proficient and reusable catalyst in the synthesis of 1,4-dihydropyridines **90** and *N*-arylquinolines **91** using environment-friendly reaction conditions. In this context, the catalyst demonstrated outstanding catalytic activity under optimized reaction conditions, resulting in the formation of desirable products **90** and **91** with high yields. Research on the catalyst's reusability showed that it could be used for five reaction cycles with a minor decrease in catalytic activity and minimal copper leakage.

**Scheme 82:**
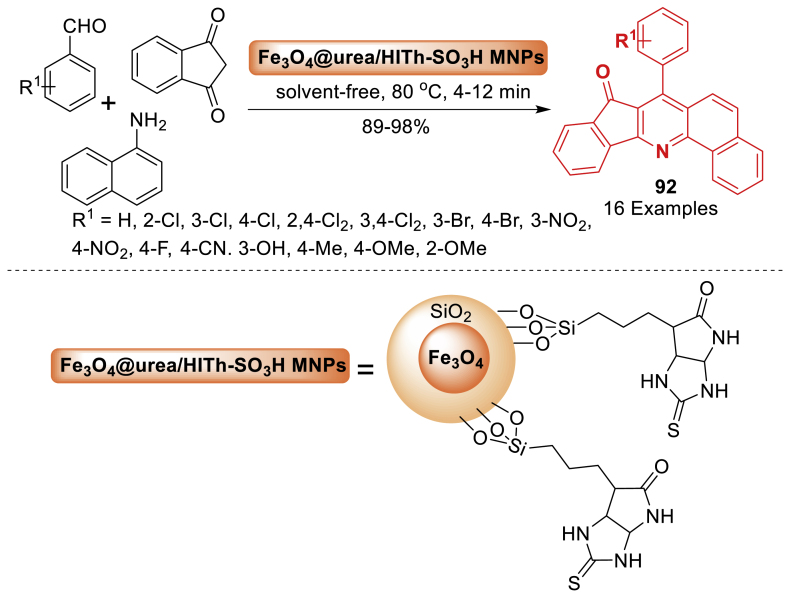
Synthesis of 7-aryl-8*H*-benzo[*h*]indeno[1,2-*b*]quinoline-8-one derivatives using Fe_3_O_4_@urea/HITh-SO_3_H

Here, Sheykhahmad et al. [127] synthesized Fe_3_O_4_@urea/HITh-SO_3_H MNPs as a solid acid magnetic nanocatalyst. Further novel magnetic nanocatalyst was characterized and effectively used to facilitate the one-pot synthesis of 7-aryl-8*H*-benzo[*h*]indeno[1,2-*b*]quinoline-8-one **92** as mentioned in Scheme 82. This process was accomplished through a three-component reaction involving 1,3-indane dione, aldehyde, and 1-naphthylamine/1,3-dimethyl-6-aminouracil under solvent-free conditions at 80 °C. The nanocatalyst's magnetic properties allow it to be separated using an external magnetic field and reused for at least six cycles without a significant loss in catalytic activity.

**Scheme 83:**
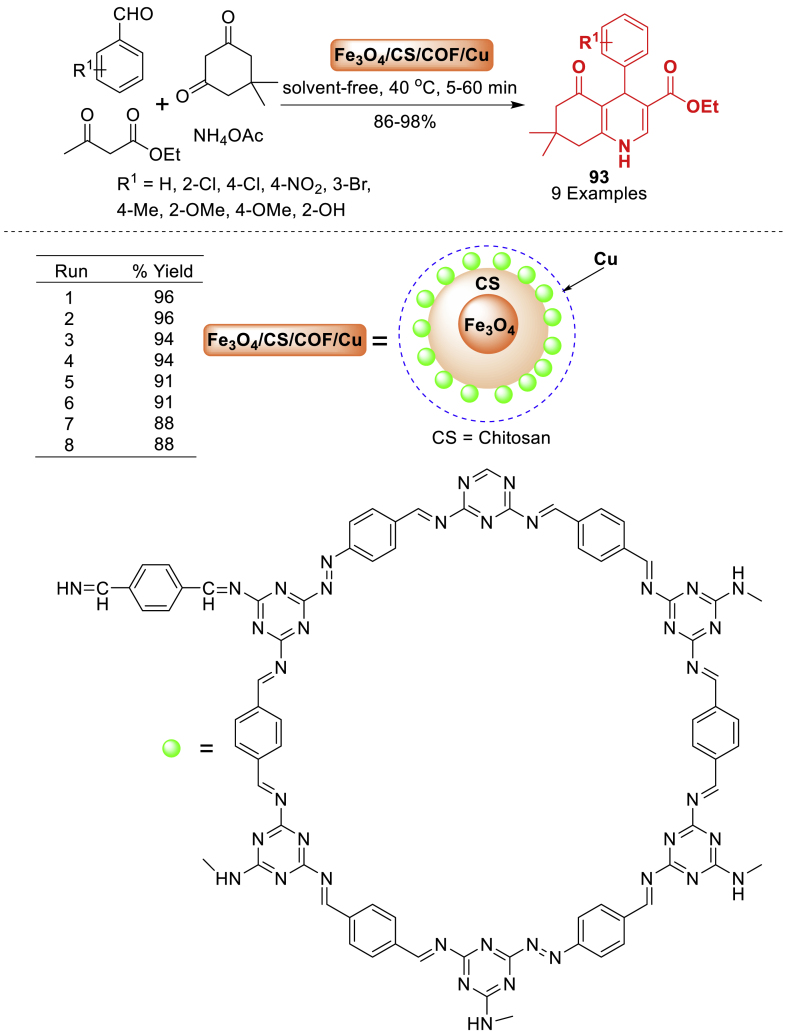
Synthesis of polyhydroquinoline derivatives.

A new nanocomposite, Fe_3_O_4_/CS/COF/Cu, was fabricated by Rafiee et al. [128] through the modifying Fe_3_O_4_ with CS, attaching a melamine-rich COF to Fe_3_O_4_/CS, and reacting with Cu(NO_3_)_2_.3H_2_O. The material Fe_3_O_4_/CS/COF/Cu has a high specific surface area and supermagnetic properties, which make it an excellent catalyst for organic processes. This nanosystem was utilized as a highly effective and reusable catalyst for the eco-friendly and easy formation of biologically active polyhydroquinoline derivatives **93** through the unsymmetric Hantzsch reaction without the need of solvents (Scheme 83). The Fe_3_O_4_/CS/COF/Cu catalyst shows excellent reactivity and selectivity in synthesizing several polyhydroquinoline derivatives **93** under mild conditions.

**Scheme 84:**
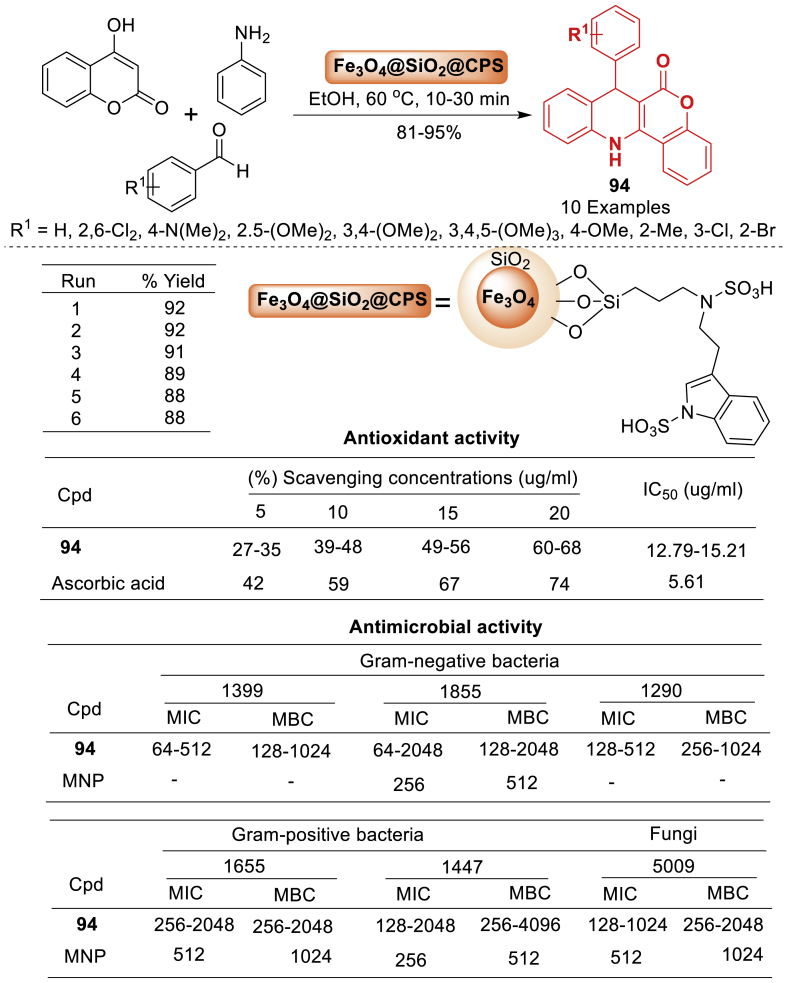
Synthesis of chromeno[4,3-*b*]quinolin-6-one derivatives.

In another work of Shirzaei and co-workers [129] synthesized new derivatives of chromeno[4,3-*b*]quinolin-6-one **94** with a new SO_3_H-tryptamine supported on Fe_3_O_4_@ SiO_2_@ CPS, which could be recycled as an effective magnetic nanocatalyst as illustrated in Scheme 84. The antimicrobial (antibacterial and antifungal) function of magnetic nanocatalysts and derivatives **94** synthesized was assessed using MIC, MBC, and MFC values. Furthermore, all the compounds showed good biological activities as mentioned in Scheme 84.

**Scheme 85:**
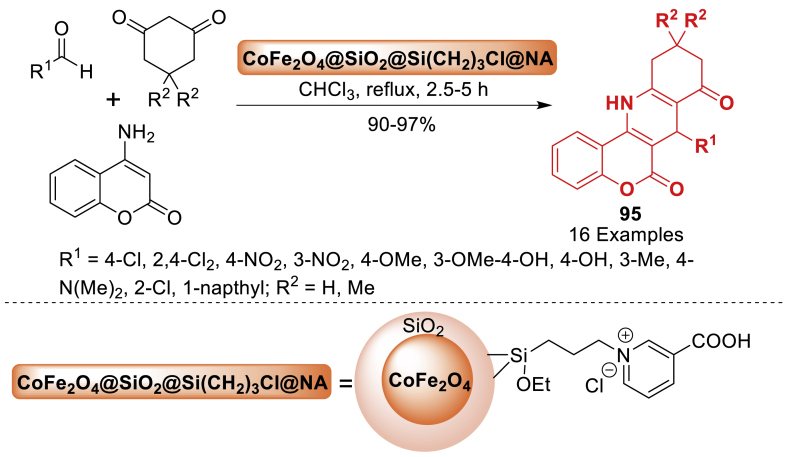
Synthesis of chromeno[3,4-*b*]quinolines using CoFe_2_O_4_@SiO_2_@Si(CH_2_)_3_Cl@NA

A three-component reaction involving aldehydes, dimedone or 1,3-cyclohexadione, and 4-aminocoumarin was used to develop a one-pot synthesis of substituted chromeno[3,4-*b*]quinoline derivatives **95** by Nikpassand et al. [130]. This reaction was carried out in the presence of nicotinic acid-supported cobalt ferrite [CoFe_2_O_4_@SiO_2_@Si(CH_2_)_3_Cl@NA] as a novel magnetic catalyst in chloroform under reflux conditions. Furthermore, the catalyst could be recycled up to five times without significantly losing its catalytic activity by simply recovering it by magnetic separation. The starting substrates having electron donating and electron withdrawing groups played very well for the present reaction condition (Scheme 85).

**Scheme 86:**
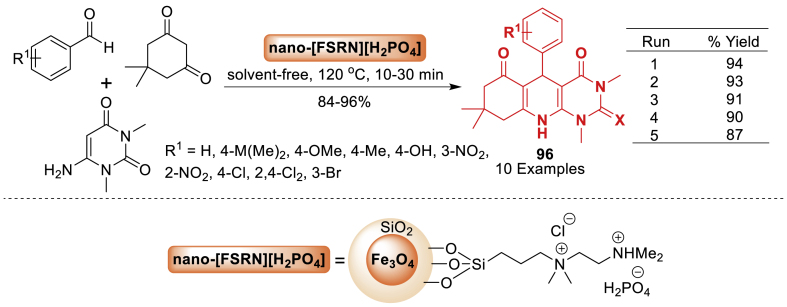
Preparation of pyrimido[4,5-*b*]quinolines.

Dianat’s research group [131] developed nano-[Fe_3_O_4_@-SiO_2_@R-NHMe_2_][H_2_PO_4_] (nano-[FSRN][H_2_PO_4_]), a novel organic-inorganic hybrid magnetic nanomaterial. Then, employing nano-[FSRN][H_2_PO_4_] as a dual-functional catalyst in a solvent-free one-pot multi-component reaction of 6-amino-1,3-dimethyluracil with arylaldehydes and dimedone, an important class of uracil-bearing heterocycles known as pyrimido[4,5-*b*]quinolines **96** was fabricated. Total 10 examples were prepared in 84–96 % yields. Some benefits of the work include high yields, short reaction times (10–30 min), the catalyst's magnetic recoverability, the absence of column chromatography required for the product purification, and good adherence to green chemistry guidelines (Scheme 86).

**Scheme 87:**
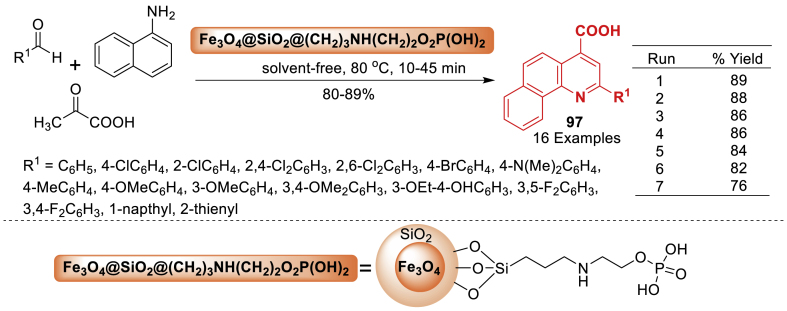
Synthesis of benzo-[*h*]quinoline-4-carboxylic acids.

Zolfigol et al. [132] fabricated Fe_3_O_4_@SiO_2_@(CH_2_)_3_NH(CH_2_)_2_O_2_P(OH)_2_ as a novel, recoverable nanocatalyst with magnetic characteristics. Benzo-[*h*]quinoline-4-carboxylic acids **97** synthesized using a three-component reaction involving aryl aldehydes, naphtylamine, and pyrovic acid without the presence of a solvent. Total 16 examples were prepared in 80–89 % yield by the author as shown in Scheme 87. After six runs, the catalyst was recycled and utilized again without a discernible drop in yield or reaction time. A range of substituted benzaldehyde were tested and all gave positive results for the current transformation.

**Scheme 88:**
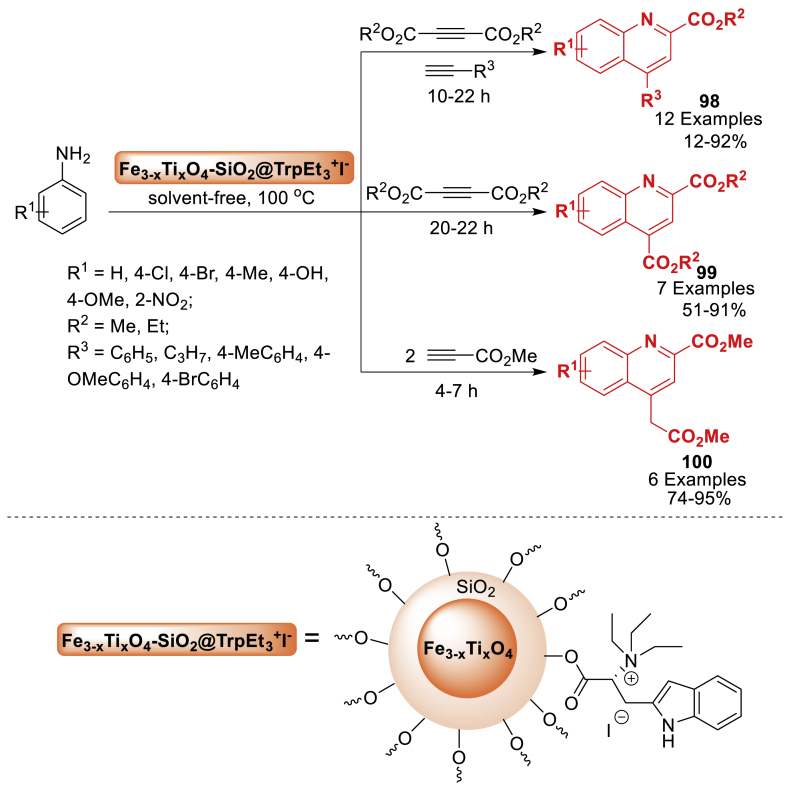
One-pot synthesis of diversified quinoline-2-carboxylates, 6-substituted quinolinedialkyl-2,4-dicarboxylates and 6-substituted 4-(2-methoxy-2-oxoethyl)quinoline-2-methylcarboxylates.

In 2021, Yielzoleh’s research group [133] synthesized a collection of various substituted quinolone-2-carboxylates **98**–**100** using novel nano catalyst system. The current reaction included one-pot multicomponent reactions of aromatic amines, dialkyl acetylene dicarboxylates, and terminal alkenes/ketones; pseudo three-component reactions of anilines and dialkyl acetylene dicarboxylates; and pseudo three-component reactions of anilines and methyl propiolate under solvent-free conditions at 100 °C. It is noteworthy to mention that the protocol's key features include the synthesis of a wide variety of substituted quinolines **98**–**100** using a simple method from basic substrates without the need of solvents, the use of different functional substrates, easy separation of the nano promoter with an external magnet, regioselectivity in the cascade annulation process, and the ability to recover and reuse the nano composite up to three times without a notable loss in activity (Scheme 88).

**Scheme 89:**
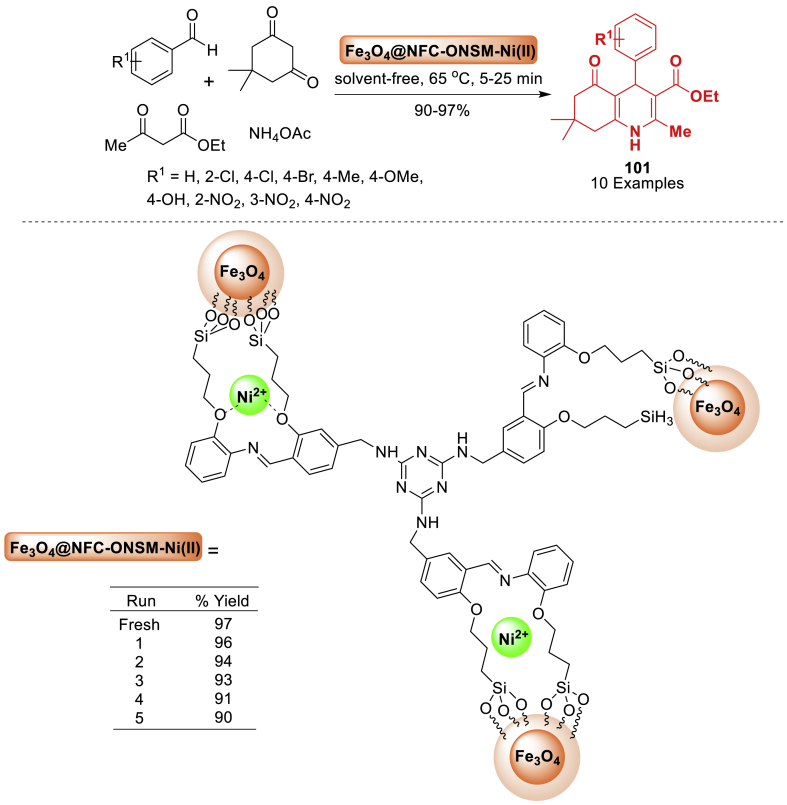
Synthesis of polyhydroquinolines by using Fe_3_O_4_@NFC@ONSM-Ni(II)

Under this research, a heterogeneous magnetically recoverable nanocomposite, Fe_3_O_4_@NFC@ONSM-Ni(II), was fabricated by Chamani et al. [134]. It was utilized as an effective catalyst for the one-pot solvent-free synthesis of polyhydroquinoline derivatives **101** *via* the Hantzsch reaction (Scheme 89). This catalyst demonstrated a significant advantage over previously reported catalysts due to optimal circumstances, short reaction time (5–25 min), high efficiency, decreased catalyst load, and rapid recovery of the magnetic catalyst. Furthermore, the effects of Fe_3_O_4_@NFC@ONSM-Ni(II) nanoparticles on the *in vitro* proliferation of human leukemia cell lines (k562) and human breast cancer cells (MDA-MB-231) were studied. MTT and Hochest assays revealed that the nanoparticles could effectively limit the proliferation of these cancer cells in a time- and concentration-dependent manner.

**Scheme 90:**
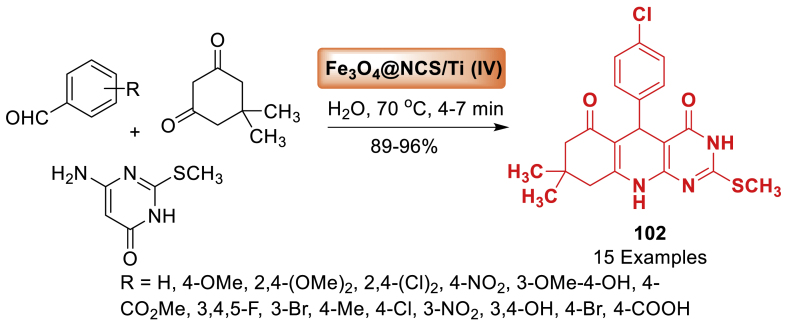
Fe_3_O_4_@NCs/Ti(IV)-catalysed synthesis of fused quinoline derivatives.

Next, in 2022, Mirjalili et al. [135] presented a straightforward and effective method for synthesizing pyrimido [4,5-b] quinoline derivatives **102** as illustrated in Scheme 90. Pyrimido [4,5-*b*] quinolines **102** were constructed using a three-component one-pot reaction involving 6-amino-2-(methylthio)pyrimidin-4(3*H*)-one, dimedone, and substituted aldehydes. This reaction comnpleted in water at 70 °C, utilizing Fe_3_O_4_@NCs/Ti(IV) as a magnetic catalyst. This approach has several benefits, including high yield (89–96 %), quick reaction time (4–7 min), simple work-up, catalyst recyclability, and environmental friendliness.

**Scheme 91:**
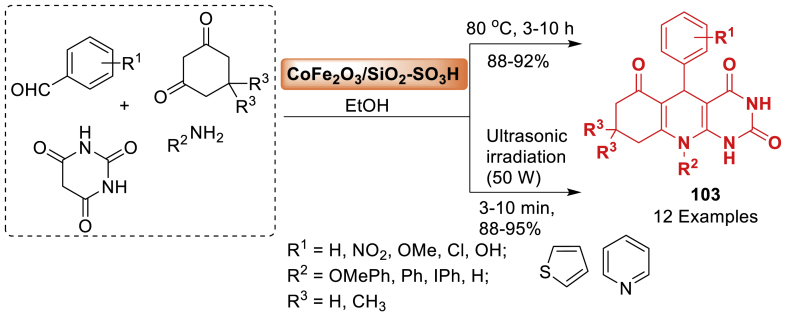
Green synthesis of dihydropyrimido[4,5-*b*]quinolinetriones.

An effective solid acid catalyst, consisting of cobalt ferrite nanoparticles covered with silica and functionalized with sulfonic acid groups (CoFe_2_O_4_/SiO_2_-SO_3_H), was produced by the research group of Moradi [136] (Scheme 91). The CoFe_2_O_4_ nanoparticles (NPs) were synthesized using the co-precipitation method. The CoFe_2_O_4_/SiO_2_-SO_3_H composite was effectively employed in the four-component reaction of barbituric acid, dimedone, amines (or ammonium acetate), and benzaldehyde derivatives. This reaction resulted in the synthesis of dihydropyrimido[4,5-*b*]quinolinetriones **103** under both heat and ultrasonic conditions. The ultrsonic irradiation method significantly reduced the reaction time and obtained the product **103** in 88–95 % yield.

**Scheme 92:**
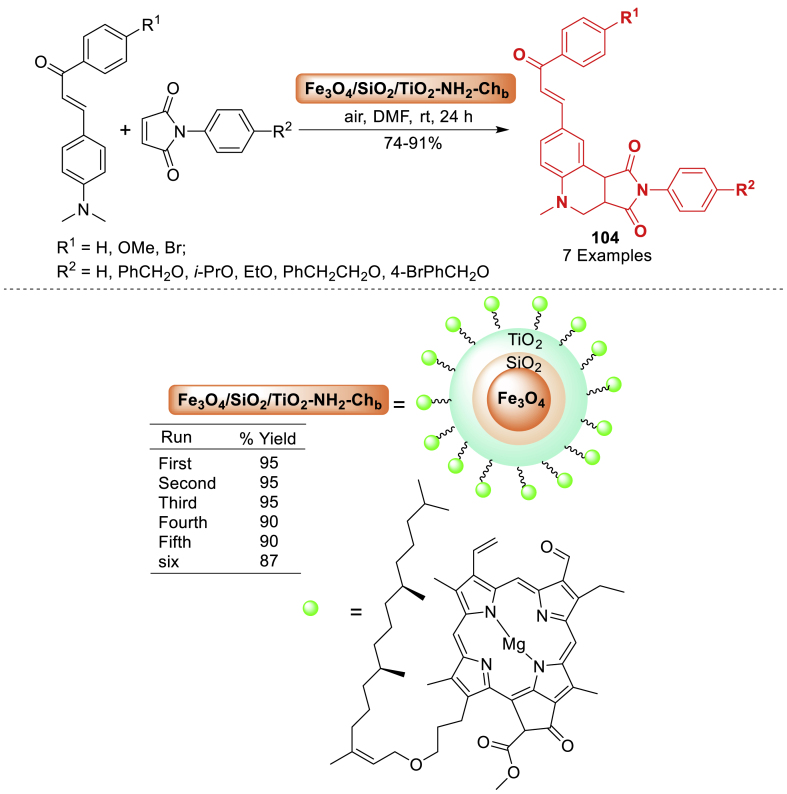
Synthesis of tetrahydroquinolines.

In 2023, the research group of Safa [137] used a photoinduced electron transfer (PET) process to fabricate conjugated derivatives of tetrahydroquinoline using a magnetic titanium dioxide-based (Fe_3_O_4_/SiO_2_/TiO_2_) photocatalyst (Scheme 92). In order to enhance light harvesting capabilities, terminal amine (Fe_3_O_4_/SiO_2_/TiO_2_-NH_2_-Ch_b_) was added to 3-aminopropyltriethoxysilane (APTES) as a coupling agent, which immobilized chlorophyll *b* as a naturally visible light-sensitive compound on the surface of magnetic titanium dioxide. Chlorophyll *b* modified magnetic titanium dioxide was extremely active under visible-light irradiation toward the reaction of (*E*)-3-[4-(dimethylamino)phenyl)]-1-arylprop-2-en-1-one and with 1-aryl-1*H*-pyrrole-2,5-dione to afford novel tetrahydroquinoline derivatives **104** with conjugated structures in high yields (74–91 %) at ambient temperature and air. Furthermore, the magnetic characteristic allowed for easy recovery of the photocatalyst and increased its reusability up to three times.

**Scheme 93:**
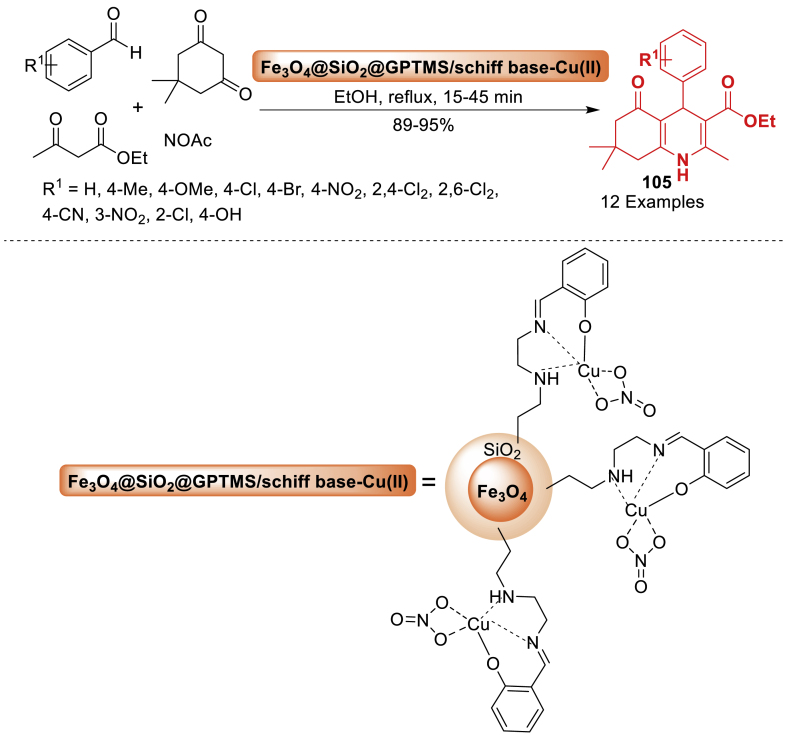
One-pot synthesis of polyhydroquinoline.

In the current study, Ramazani and co-workers [138] demonstrated an eco-friendly and efficient process for coating of copper (II) on the surface of Schiff base complex immobilized on Fe_3_O_4_@SiO_2_ MNPs to fabricate a Core/shell nanostructure Fe_3_O_4_@SiO_2_@GPTMS/Schiff base-Cu(II) nanocatalyst. In particular, the prepared nanocatalyst exhibits strong activity in the one-pot synthesis of polyhydroquinoline derivatives **105** of a wide range of aldehydes, dimedone, ethyl acetoacetate, and ammonium acetate (Scheme 93). The experimental evidence shows that the proposed technique has many advantages, including strong thermal stability of the catalyst, simple workup, environmental-friendly, atom economy, easy reaction conditions, and without column chromatographic separation.

**Scheme 94:**
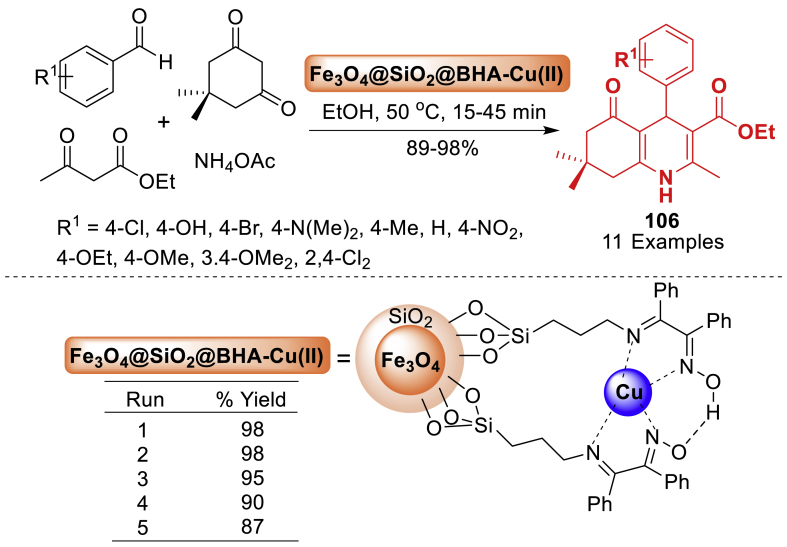
Synthesis of polyhydroquinolines derivatives.

A magnetically recoverable heterogeneous Fe_3_O_4_@SiO_2_@BHA-Cu(II) nanocomposite was fabricated by Beiranvand et al. [139] immobilizing a new Cu(II) complex on the nanoparticles as illustrated in Scheme 94. The catalytic activity of nanocatalyst in the synthesis of polyhydroquinoline derivatives **106** was studied. This recyclable magnetic nanocatalyst offers great advantages such as a quick reaction time, high atom economy, ease of setup, mild reaction conditions, and excellent product yields. All the substituted benzaldehydes were well tolerated towards the present method to afford the quinoline derivatives.

**Scheme 95:**
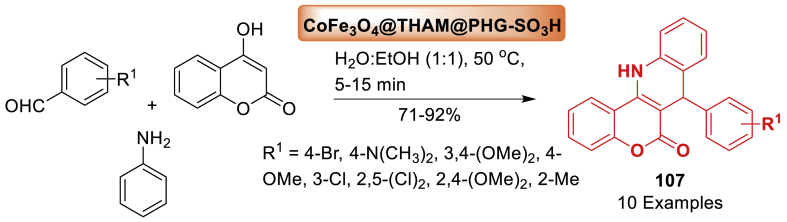
Synthesis of chromeno[3,4-*b*] quinoline-6-ones by the catalysis of nanocatalyst.

Very recently (2024), Hazeri’s research team [140] fabricated a novel magnetic nanocatalyst (Scheme 95). They achieved magnetic nanocatalyst by subjecting CoFe₂O₄ magnetic nanoparticles to a treatment using chlorosulfonic acid, tris(hydroxymethyl)aminomethane (THAM), 1,2-dichloroethane, and phloroglucinol (PHG). The nanocatalyst's catalytic efficiency was assessed in the synthesis of chromeno[3,4-b] quinoline-6-ones **107** through the reaction of aromatic aldehydes, 4-hydroxycoumarin, and aniline. Total 10 examples (**107**) were synthesized in 71–92 % yields by the author.

**Scheme 96:**
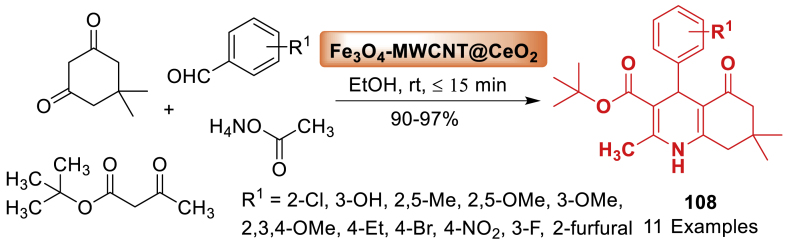
Formation of *tert*-butyl-quinoline analogues.

In the follwing year, a simple impregnation protocol was used to synthesize Fe_3_O_4_-MWCNT@CeO_2_ nano-composite by Maddila *et al* [141] (Scheme 96). Next, the synthesis of novel *tert*-butyl-quinoline **108** analogues *via* a multi-component condensation containing substituted aldehydes, dimedone, 3-butylacetoacetate, and NH_4_OAc to evaluate the catalytic performance of the Fe_3_O_4_-MWCNT@CeO_2_ nano-composite. Their large specific surface area, porosity, unique exposed surfaces, and stability all contribute to their effectiveness. This methodology offers several advantages such as excellent yields, green method and short reaction time.

**Scheme 97:**
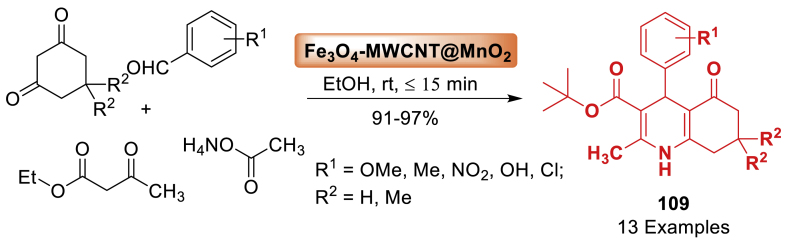
Fe_3_O_4_-MWCNT@MnO_2_-catalysed formation of tethered quinoline derivatives.

In another study, Maddila et al. [142] investigated the catalytic capabilities of a nano-composite material consisting of manganese oxide (MnO_2_) supported composite of iron oxide and multi-walled carbon nanotubes (Fe_3_O_4_-MWCNT). Next, the catalytic efficiency of the Fe_3_O_4_-MWCNT@MnO_2_ nano-composite was evaluated for the synthesis of hexahydro-quinoline analogues **109** using a multi-component condensation process involving substituted aldehydes, cyclohexadione, ethylacetoacetate, and NH_4_OAc. The catalyst demonstrated efficacy due to its significant specific surface area, stability, porosity, and unique exposed surfaces. Notably, this method employed ethanol as a sustainable solvent, cost effective and environmental safe nanocomposite (Scheme 97).

**Scheme 98:**
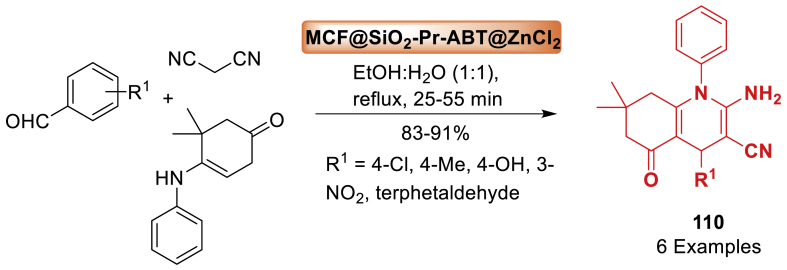
MNPs-catalysed synthesis of *N*-arylquinoline.

The current study Mobinikhaledi’s research team [143] involved the synthesis of silica-coated hybrid manganese doped cobalt ferrite nanoparticles, with Zn complexed on their surface (Scheme 98). Thereafter, the catalytic performance of these fabricated magnetic nanoparticles (MNPs) were investigated in the synthesis of *N*-arylquinoline derivatives **110**. This approach offers high product yield (83–91 %), short reaction time (25–55 min), straightforward purification, and convenient separation of the catalyst. Moreover, the catalyst's ability to be reused for five periods did not result in a notable decline in its activity.

**Scheme 99:**
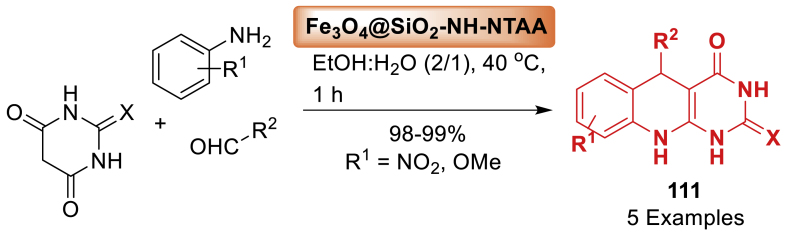
Green synthesis of pyrimido [4,5]quinolone-2,4-diones.

Under this work, the synthesis and performance of Fe_3_O_4_@SiO_2_-NH-NTAA has been described by Nikmanesh et al. [144] as depicted in Scheme 99. The catalytic efficiency, ease of separation, recoverability, and reusability of the Fe_3_O_4_@SiO_2_-NH-NTAA magnetic nano-catalyst were evaluated in the one-pot synthesis of pyrimido [4,5]quinolone-2,4-diones **111**. The Fe_3_O_4_@SiO_2_-NH-NTAA catalyst demonstrated excellent performance with a reaction yield of 98–99 % and a catalyst recovery efficiency of over 98 %.

**Scheme 100:**
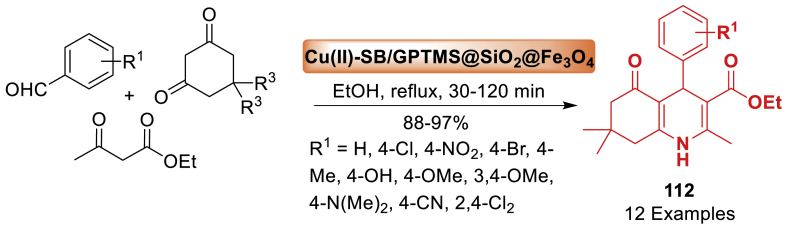
Synthesis of polyhydroquinoline.

In the same year, Ramazani et al. [145] successfully fabricated a durable and reusable compound called a Schiff base complex of copper (Scheme 100). This compound is attached to magnetic nanoparticles with a core-shell structure [Cu(II)-SB/GPTMS@SiO_2_@Fe_3_O_4_]. Next, the nanocatalyst demonstrated excellent catalytic efficiency in the one-pot synthesis of polyhydroquinoline derivatives **112** through the Hantzsch reaction. This reaction involved the use of dimedone, ethyl acetoacetate, ammonium acetate, and different aldehydes. The products **112** are obtained with yields ranging from 88 % to 97 %.

### 2.3 Hantzsch Synthesis mechanisms

**Fig. 3:**
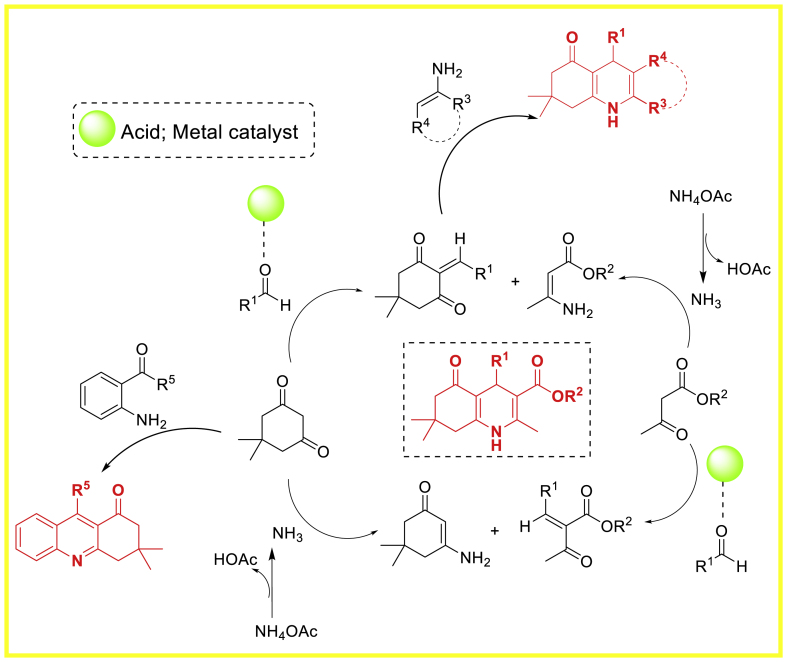
Generalized mechanistic route for the synthesis of quinoline.

Fig. 3 illustrates the overall mechanism of the Hantzsch Synthesis, which is used to produce quinoline derivatives. Initially, the reaction can be observed as progressing through a Knoevenagel condensation product to generate the intermediate, further the catalyst is facilitating the condensation of activated aldehydes with active methylene compounds (dimedone or ethyl acetoacetate) to afford an *α,β*-unsaturated intermediate. The catalyst may be in the form of acid catalysts, including Lewis acids (transition metals) or Brønsted-Lowry acids (acidic protons). Importantly, in the case of basic catalysts, the basic sites remove the acidic proton from methylene compounds to access the Knoevenagel condensation. This condensation reaction occurs between the resulting carbanion and the activated aldehyde, ultimately leading to the formation of the Knoevenagel adduct. In addition, the second intermediate, an ester enaminone, is formed through the condensation of the activated carbonyl group of dimedone or ethyl acetoacetate diketones with ammonia. The catalyst is facilitating the Michael addition reaction, which involves the intramolecular cyclization of enaminone and *α,β*-unsaturated intermediates. Finally, this reaction ultimately leads to the formation of the quinoline product.

### 2.4 Comparison of various MNPs

To assess the effectiveness of the reviewed reports, we compared the results obtained from the synthesis of diversity-oriented quinolines using benzaldehyde, diketoester, 5,5-dimethylcyclohexane-1,3-dione and ammonium acetate as the model reactant, as detailed in Table 1.

**Table 1:** Analysis of various types of MNPs on the synthesis of quinoline derivatives.


**S. No.**	**Catalyst**	**Time (min)**	**Yield (%)**	**Ref.**	**Scheme**
1.	Fe_3_O_4_	6	89	[48]	3
2.	Fe_3_O_4_	5	94	[50]	5
3.	NiFe_2_O_4_	3	90	[56]	11
4.	Co_3_O_4_	22	91	[57]	12
5.	BiFeO_3_	3	97	[59]	14
6.	Fe_3_O_4_	60	94	[62]	17
7.	nano-*γ*-Fe2O3-SO3H	65	98	[67]	22
8.	NZFS-Prs	60	91	[69]	24
9.	Ni-Cu-MgFe_2_O_4_	2	94	[75]	30
10.	AIL-SCMNPs	10	92	[77]	32
11.	NiFe_2_O_4_@SiO_2_-H_14_[NaP_5_W_30_O_110_]	10	94	[79]	34
12.	Fe_3-x_Ti_x_O_4_-SO_3_H	25	85	[81]	36
13.	Magnetic dextrin	27	70	[83]	38
14.	CoFe_2_O_4_@Pr	400	91	[84]	39
15.	Fe_3_O_4_@Ca(HSO_4_)_2_	3	94.6	[85]	40
16.	Hercynite@sulfuric acid	30	98	[89]	44
17.	Fe_3_O_4_@MgO	10	94	[92]	47
18.	*γ*-Fe_2_O_3_/Cu/cellulose	20	93	[93]	48
19.	Fe_3_O_4_-DETA-Cu(II)	50	96	[95]	50
20.	Fe_3_O_4_@sucrose	30	93	[100]	55
21.	Fe_3_O_4_@melamine@SO_3_H	5	85	[101]	56
22.	SBA-15@AMPD-Co	65	96	[121]	76
23.	Fe_3_O_4_@SiO_2_@Si(CH_2_)_3_@HMTA-Cu	4	90	[122]	77
24.	[Fe_3_O_4_@SiO_2_@(CH_2_)_3_Py]HSO_4_^-^	90	98	[124]	79
25.	MnFe_2_O_4_@SiO_2_-Pr-NH@BSTA@Cu(OAc)_2_	30	94	[126]	81
26.	Fe_3_O_4_/CS/COF/Cu	15	96	[128]	83
27.	Fe_3_O_4_@NFC@ONSM-Ni(II)	10	97	[134]	89
28.	Fe_3_O_4_@SiO_2_@GPTMS/Schiff base-Cu(II)	30	94	[138]	93
29.	Fe_3_O_4_@SiO_2_@BHA-Cu(II)	25	94	[139]	94

### 2.5 Challenges

Although MNPs have various advantages in synthesizing quinoline derivatives, there are limitations and obstacles in using MNPs as a catalyst. Magnetic nanoparticles (MNPs) exhibit a substantial surface energy, leading to the occurrence of agglomeration or clustering. This reduces their effective surface area and efficiency as catalysts. Intermittently, magnetic fields can interrupt the catalytic activity of MNPs, or the catalytic sites may become deactivated throughout the reaction. Functionalizing magnetic nanoparticles (MNPs) with specific catalytic sites can be complex and time-consuming, requiring multiple steps and careful control of reaction conditions. Inadequate handling of nanoparticles, especially during their manufacturing, usage, and disposal, can result in substantial environmental and health risks. By incorporating modifications to the surface, enhancing magnetic properties, streamlining synthesis techniques, and following environmentally friendly chemistry principles, these challenges can be effectively overcome, making magnetic nanoparticles (MNPs) a viable and efficient option for catalysing organic reaction.

### 2.6 Summary and outlook

**Fig. 4:**
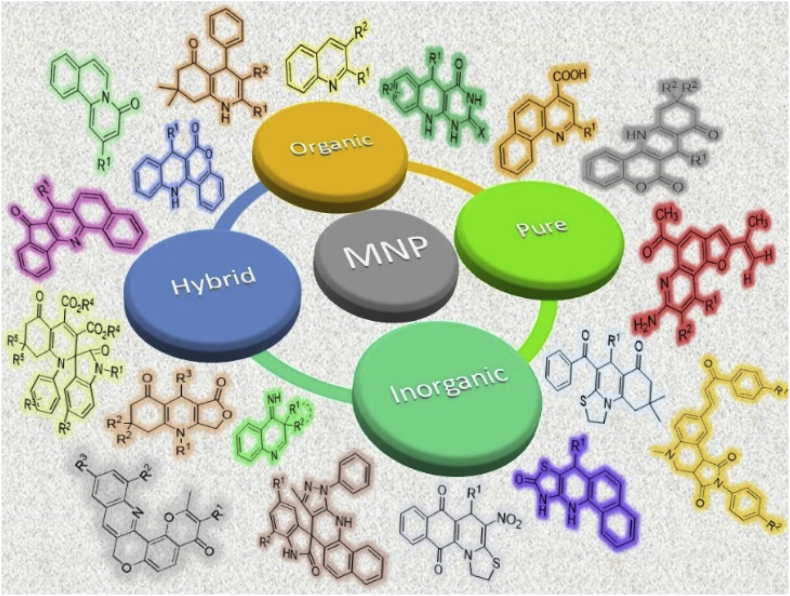
A summary of molecules synthesized through the catalysis of magnetic nanoparticles.

There has been a notable rise in the use of nanoparticles in the organic synthesis in recent years, which might be due to distinct advantages, like enhanced activity, low toxicity, extended surface area, and the ability to be retrieved and reused. The entire process of utilizing magnetic nanoparticles in the synthesis of quinoline derivatives, hold significant importance. Quinoline and its derivatives are frequently included in the chemical composition of numerous natural products and biological compounds, displaying a wide range of applications in various fields. Quinoline derivatives offer significant advantages in the fields of biological and material research, owing to their numerous advantageous characteristics. Several quinoline based bioactive framework such as polyhydroquinolines, tetrahydroquinolines, pyrimido[4,5-*b*]quinolines, chromeno[3,4-*b*]quinolines, hexahydroquinoline, benzo[*g*]thiazolo[3,2-*a*]quinolones, 4-iminoquinolines, thiazoloquinolines, furo[2,3-*f*]quinoline and spiro[indoline-3,2′-quinolines] have been synthesized by utilizing the magnetic nano particles as a catalyst. This review has the potential to offer fundamental insights of environment-friendly catalyst for synthesizing novel quinoline-based heterocyclic molecules. Fig. 4 illustrates a visual summary of this review.

### 2.7 Future scope

It is well understood *via* previous investigations that the full potential of magnetic nanoparticles for synthesizing quinoline based molecular structures has not been fully investigated. The potential applications of magnetic nanoparticles in multicomponent reactions and other contemporary chemistry for the synthesis of quinoline scaffolds have been nicely discussed in this review article. Exploring magnetic nanoparticles to their full potential in synthesizing quinoline derivatives has the potential to yield far more promising and commendable outcomes. Based on our summarized findings, it is suggested that highly effective magnetic nanoparticles can have broader applications in the domain of quinoline-based frameworks. The scientific community working in nano and organic chemistry will gain more clear understanding to carry forward these research domains in a more desirable as well as eco-friendly manner.

## CRediT authorship contribution statement

**Vaishali:** Writing – original draft, Conceptualization. **Shubham Sharma:** Writing – review & editing. **Pooja Sharma:** Formal analysis. **D.K. Das:** Writing – review & editing. **Vinod K Vashistha:** Visualization, Software. **Jitender Dhiman:** Methodology, Investigation. **Rachna Sharma:** Writing – review & editing, Writing – original draft. **Rajesh Kumar:** Formal analysis, Data curation. **Man vir Singh:** Resources. **Yogendra Kumar:** Writing – review & editing, Writing – original draft.

## Declaration of competing interest

The authors declare that they have no known competing financial interests or personal relationships that could have appeared to influence the work reported in this paper.
